# CalDyn: an advanced high-throughput automated algorithm for monitoring intracellular calcium dynamics and electrophysiological signals in patient-derived cells

**DOI:** 10.1093/bib/bbag602

**Published:** 2026-11-07

**Authors:** Yvonne Sleiman, Georges El Achkar, Sarah Colombani, Eric Estève, Jean-Baptiste Reisqs, Marie Kervella, Lamia Goual, Elisa Bounasri, Maria João Ferreira, Michael Cupelli, Angelo G Torrente, Jérôme Thireau, Pedro F Costa, Mohamed Boutjdir, Alain Lacampagne, Antoine Muchir, Pascal Seyer, Hélène Delanoë-Ayari, Albano C Meli

**Affiliations:** PhyMedExp, Univ Montpellier, INSERM, CNRS, Montpellier, France; Cardiovascular Research Program, VA New York Harbor Healthcare System, New York, United States; International Center in Fundamental and Engineering Thermophysics, Tianjin University of Commerce, Tianjin, China; Energy and Thermo-Fluid group, Lebanese International University, Bekaa, Lebanon; PhyMedExp, Univ Montpellier, INSERM, CNRS, Montpellier, France; PhyMedExp, Univ Montpellier, INSERM, CNRS, Montpellier, France; PhyMedExp, Univ Montpellier, INSERM, CNRS, Montpellier, France; Cardiovascular Research Program, VA New York Harbor Healthcare System, New York, United States; PhyMedExp, Univ Montpellier, INSERM, CNRS, Montpellier, France; Institute of Myology, Center of Research in Myology, Sorbonne University, Inserm, Paris, France; PhyMedExp, Univ Montpellier, INSERM, CNRS, Montpellier, France; PhyMedExp, Univ Montpellier, INSERM, CNRS, Montpellier, France; MicroBrain Biotech S.A.S., Marly Le-Roi, France; PhyMedExp, Univ Montpellier, INSERM, CNRS, Montpellier, France; Cardiovascular Research Program, VA New York Harbor Healthcare System, New York, United States; Department of Medicine, Cell Biology and Pharmacology, State University of New York Downstate Health Sciences University, New York, NY, United States; IGF, Univ Montpellier, INSERM, CNRS, Montpellier, France; PhyMedExp, Univ Montpellier, INSERM, CNRS, Montpellier, France; Biofabics, Porto, Portugal; Cardiovascular Research Program, VA New York Harbor Healthcare System, New York, United States; Department of Medicine, Cell Biology and Pharmacology, State University of New York Downstate Health Sciences University, New York, NY, United States; Department of Medicine, New York University Grossman School of Medicine, New York, United States; PhyMedExp, Univ Montpellier, INSERM, CNRS, Montpellier, France; Institute of Myology, Center of Research in Myology, Sorbonne University, Inserm, Paris, France; PhyMedExp, Univ Montpellier, INSERM, CNRS, Montpellier, France; University of Lyon, CNRS, Institut Lumière Matière, Villeurbanne, France; PhyMedExp, Univ Montpellier, INSERM, CNRS, Montpellier, France; Biocampus, Univ Montpellier, CNRS, INSERM, Montpellier, France

**Keywords:** intracellular Ca^2+^ handling, arrhythmias, human induced pluripotent stem cells (hiPSCs), cardiomyocytes, neurons, action potential, optical mapping, electrophysiology, and analytical techniques

## Abstract

**Background:**

Quantitative analysis of intracellular calcium and electrophysiological signals is challenging when signals exhibit baseline drift, low-amplitude events, irregular waveforms, or multicomponent transients. We developed CalDyn, a MATLAB-based graphical application for automated peak detection, waveform characterization, event classification, batch processing, quality control and data export.

**Methods:**

CalDyn combines Savitzky–Golay filtering, LOESS-assisted derivative detection, optional baseline correction, waveform-boundary fitting and rule-based peak classification. It was evaluated on calcium recordings (healthy and disease-associated hiPSC-derived cardiomyocytes, hiPSC-derived neurons, primary skeletal myotubes), current-clamp action potentials and cardiac optical-mapping signals. Outputs were compared with PeakInspector, Clampfit and BV Workbench using paired and Bland–Altman analyses. Detection was assessed against 142 expert-annotated events and synthetic signals of known timing and amplitude.

**Results:**

CalDyn achieved 94.4% sensitivity, 89.9% precision and an F1 score of 0.92. Performance peaked for healthy cardiomyocytes, paced myotubes and action potentials, with lower recall in arrhythmic cardiomyocytes and variable precision in neurons. CalDyn detected candidate low-amplitude diastolic and multicomponent Ca^2+^ events in arrhythmic cardiomyocytes, although the biological identity of CalDyn-only events was not independently confirmed. Comparisons with software revealed parameter-specific systematic differences, notably for rise rates and classifications. Action-potential measurements showed limited mean bias but non-negligible limits of agreement. In synthetic signals, amplitude error increased with noise and uncorrected drift, and timing error with low sampling rates. Detection remained stable across moderate parameter variation.

**Conclusions:**

CalDyn provides an accessible, reproducible and versatile workflow for automated analysis of heterogeneous calcium and electrophysiological signals. With appropriate quality control, it offers a robust alternative to modality-specific tools.

## Introduction

Action potential (AP) evoked intracellular calcium (Ca^2+^) variations serve as a ubiquitous transduction signal in eukaryotic cells, playing a pivotal role in various cellular processes. At the cellular membrane level, increased depolarizing current or decreased repolarizing current could lead to abnormal AP shape, including aberrant electrical activity (cardiac arrhythmia or neuronal hyperexcitability). As a direct consequence, intracellular Ca^2+^ signaling could be disrupted, leading to physiological dysfunction, as observed in heart failure and Alzheimer’s disease, which are linked to aberrant intracellular Ca^2+^ release [1].

The advancement of human induced pluripotent stem cell technology (hiPSC), particularly in generating hiPSC-derived cardiomyocytes (hiPSC-CMs) and neurons (hiPSC-NRs), holds great promise for investigating the genotype–phenotype relationship within a patient’s cellular and molecular context [1]. This technology allows for modeling complex diseases to decipher the cellular and molecular mechanisms and even to evaluate potential therapeutic candidates through the acquisition and analysis of gold standard electrophysiological and Ca^2+^ transient parameters.

For analysis, workflows built on open-source solutions often necessitate a high degree of user expertise and manual intervention for accurate analysis of voltage signals or intracellular Ca^2+^ events. Furthermore, the majority of current analytical methods are optimized for adult cells that display consistent AP shape or Ca^2+^ release patterns, restricting their effectiveness when applied to myocytes and neurons in varying shapes, sizes, and phenotypes [2].

Current methods may not be well-suited for hiPSC-NRs, which often display irregular intracellular Ca^2+^ handling involving the ryanodine and inositol triphosphate receptors (RyR and IP3R, respectively). Some solutions, such as Sequential IMaging Analysis called SIMA [3], an analytical tool for Ca^2+^ peak screening [4], CaImAn [5] and CalTrack [6], aim to streamline the preprocessing of cellular data and facilitate the extraction and analysis of intracellular Ca^2+^ transients within heterogeneous populations of neurons and hiPSC-CMs. PeakInspector is a Python-based application designed for the interactive analysis of peak properties in time-series data and whole-cell or line scan modes. It utilizes text format data uploads [7]. While PeakInspector is user-friendly and features a pre-processing option for filtering and baseline normalization, its functionality is constrained by the duration of recordings, its compatibility with the evolving Python language and operating systems, and its proprietary nature. Crucially, These tools differ in their ability to detect and classify heterogeneous waveform components, including candidate low-amplitude or multicomponent Ca^2+^ events. However, such analytical classifications do not by themselves establish the underlying biological mechanism.

There are software and Python-based solutions available for analyzing recordings. Clampfit can automatically detect and analyze APs, making it easier to identify key events within recordings. It includes various filters (highpass, lowpass, bandpass) to remove noise from your signals, improving the clarity of your data. Sort spikes based on their characteristics, helping to differentiate between different types of APs. While powerful, it can have a steep learning curve for new users. As part of the pCLAMP suite, it can be expensive compared to some open-source alternatives. pyABF is a Python library designed for working with Axon Binary Format (ABF) files, which are commonly used in electrophysiology to store data from patch-clamp recordings (available at https://pypi.org/project/pyabf/). It integrates seamlessly with other Python libraries like NumPy and Matplotlib, allowing for powerful data analysis and visualization. However, for very large datasets, pyABF might not be as fast as some other specialized software, potentially leading to longer processing times.

BV Workbench is a comprehensive software tool designed for the analysis of optical mapping and Ca^2+^ imaging data, particularly for cardiac and neuroimaging applications. It supports various data formats, including 16-bit TIFF files, making it compatible with many imaging systems. BV Workbench can automatically detect peaks and set optimal values for filters and maps, streamlining the analysis process. As a specialized tool, BV Workbench requires a paid license, which might be a consideration for some users. While user-friendly, mastering all its features might take some time, especially for new users.

To address these limitations, we have developed CalDyn, a MATLAB-based algorithm designed for interactive, automated, high-throughput batch analysis of peak properties in time-series data on local computers. Prior to analysis, CalDyn offers data preprocessing capabilities, including baseline normalization, linear detrending, and data smoothing. This versatile tool is adept at monitoring and distinguishing highly variable intracellular Ca^2+^ release patterns over time in individual cells as well as cell clusters, including 3D organoids.

CalDyn’s utility extends beyond patient hiPSC-CMs and -NRs and skeletal muscle fibers; it can also be applied to various cell types, including fully mature cells. The algorithm is adaptable for analyzing AP signals and properties obtained from patch-clamp and optical mapping experiments. Notably, CalDyn efficiently processes extensive datasets through high-throughput analysis and generates robust statistical results in spreadsheet format. This resource streamlines both qualitative and quantitative assessments of intracellular Ca^2+^ handling in patient-derived control, transgenic, and pathological cells even for non-specialists. The processing time is swift (<1 s) for a data series. Once the parameters have been customized to meet specific requirements, the program can be effortlessly initiated in batch mode to process autonomously. It automatically saves corresponding graphs, including peak characteristics and localization, facilitating retrospective verification of results. CalDyn reveals the complex mechanisms of intracellular Ca^2+^ regulation under both physiological and pathological conditions, thereby greatly enhancing our knowledge of human-derived cell behavior.

## Material and methods

### Ethics statement

Written informed consent was obtained from all participants before collection of biological samples for hiPSC generation. The study was conducted in accordance with the principles of the Declaration of Helsinki and was approved by the Institutional Review Board of Montpellier University Hospital (protocol 2017-A01589-44). All hiPSC lines used in the present study were previously generated and characterized. These included the healthy control lines AG08C5 (RRID: CVCL_D0KP) and ROMDES [8], the patient-derived RyR2-H29D PMVT line [9], and the patient-derived LMNA p.H222P line and its gene-corrected isogenic control [10]. The line identity, donor characteristics, genotype, relationship between parental and isogenic lines, experimental use, and dataset provenance are summarized in Supplementary Table 1.

Regarding the skeletal muscle samples, quadriceps muscle biopsies were collected from a healthy adult (19-year-old male) at the Centre Hospitalier Universitaire in Montpellier, France. Donors provided informed written consent after being provided with a detailed protocol description (Authorization N° DC-2008-594).

For the Dunkin-Hartley guinea pigs’ samples, the experimental protocol was approved by the Institutional Animal Care and Use Committee (IACUC) at the VA New York Harbor Healthcare System, and animals were maintained in accordance with *The Guide for the Care and Use of Laboratory Animals* (National Institutes of Health, revised 2011) and followed the ARRIVE guidelines 2.0.

### Human induced PSC-CM differentiation

The 2D-sandwich protocol was used to differentiate the hiPSC lines into the cardiac lineage under standard conditions, as previously described [8]. Briefly, mesoderm induction was initiated by activating the Wnt/β-catenin pathway using 6 μM CHIR99021 (Tocris, #4423). Cardiac progenitor formation was subsequently achieved by inhibiting the Wnt/β-catenin pathway with 2 μM Wnt inhibitor C59 (Wnt C59) (Tocris, #5148). For the generation of a pure population of hiPSC-CMs, the culture was treated with SILAC medium supplemented with 4 mM Na^+^-lactate, without glucose and pyruvate, from day 11 to day 16, following the established protocol [11]. To promote a more mature phenotype, 0.1 μM 3,3′,5 triodo-L-thyronine (T3) hormone (Sigma, #T2877) and 1 μM dexamethasone (Sigma, #D4902) were employed for a duration of 10 days. The hiPSC-CMs were consistently maintained in culture until reaching day 60 of differentiation.

### Differentiation of human primary skeletal myoblasts

Myoblasts, which are muscle progenitor cells, were isolated and purified from healthy muscle biopsies and cultivated on collagen-coated dishes in DMEM medium supplemented with 20% fetal bovine serum (FBS), 0.1% Ultroser G (proliferation medium), as previously described [12]. For the process of cell differentiation, myoblasts reaching confluence were transferred to DMEM medium with 2% FBS (differentiation medium) and incubated for a period of 3 days. During this time, myoblasts aligned and fused together to form elongated, multinucleated myotubes that expressed specific skeletal muscle proteins.

### Human induced PSC-NR differentiation

We conducted neuronal midbrain differentiation following the protocol outlined by StemCell Technologies. The procedure began by seeding hiPSCs on Matrigel hESC-Qualified in Neural Induction Medium (StemCell Technologies, #08581) for a period of 3 weeks, with a passage carried out every 7 days. Subsequently, on day 21, the cells were dissociated and placed in Neural Progenitors Medium (StemCell Technologies, #05833) expansion medium for 14 days, with a passage performed after 1 week. The resulting neuronal progenitors were then subjected to another passage in Neural Differentiation Medium (StemCell Technologies, #100-0038) supplemented with SHH cytokine (200 ng/mL) for a week. Following this, the immature neurons were dissociated and cultured in Neural Maturation Medium (StemCell Technologies, #100-0041) to achieve mature neurons.

### CTX preparation

Purified ciguatoxin-1B (CTX-1B) was purchased from Fujifilm Wako Chemicals Europe GmbH (#038-25801). The compound was reconstituted in dimethyl sulfoxide (DMSO) to prepare a 1 μM stock solution, divided into aliquots, and maintained at −20°C until use. Immediately before treatment, the stock solutions were diluted in the experimental medium to a working concentration of 5 nM. Samples were exposed to the compounds at 37°C for 5 min in acute-exposure experiments. Corresponding vehicle controls received the equivalent final concentration of DMSO.

### Measurement of cytosolic Ca^2+^ variations under fluorescent confocal microscopy

Cytosolic Ca^2+^ variations were monitored in three different cell types (hiPSC-CMs, hiPSC-NRs, and human primary skeletal myotubes) using Fluo-4 AM (Thermo Fisher, #F14201), a cell-permeant, non-fluorescent precursor. Intracellular esterases cleave the AM group to generate the fluorescent Ca^2+^-binding dye Fluo-4. Cells loaded with Fluo-4 AM were excited at 488 nm and data were recorded under an inverted confocal laser scanning microscope (Zeiss LSM 800), through Zen (version 2.6, Zeiss) at 37°C.

Intracellular cytosolic Ca^2+^ variations in hiPSC-CMs, also known as Ca^2+^ transients, were measured and quantified as previously reported [9]. In brief, 60-day-old hiPSC-CMs were enzymatically dissociated 7 days prior, then loaded with 3 μM Fluo-4 AM. Intracellular esterases cleave the AM group to generate the fluorescent Ca^2+^-binding dye Fluo-4, which was measured after incubation in RPMI1640 medium (Thermo Fisher, #11875093) for 20 min at 37°C through Zen (version 2.6, Zeiss). After incubation, the probe was removed, and the medium replaced with Tyrode’s solution (NaCl 135 mM; KCl 4 mM; MgCl2 1 mM; HEPES 10 mM; CaCl2 1.8 mM; Glucose 10 mM; pH 7.4). Fluorescent changes were acquired using a ×63 objective (oil immersion) in line-scan mode (x-t mode, 1.53 ms per line, 512 pixels × 5000 lines). The data containing fluorescence intensity and time was exported as a CSV file to be operated on by CalDyn.

To evaluate the functional Ca^2+^ handling properties of multicellular preparation, control and mutated 2D cardiac sheets of previously characterized patient-derived hiPSC-CMs [10] were subjected to live-cell fluorescent Ca^2+^ imaging. Culture media was removed, and the cardiac sheets were gently washed with standard Tyrode’s solution. The cells were then loaded with the intracellular Ca^2+^ indicator 5 μM Fluo-4 AM dissolved in RPMI and incubated for 30 min at 37°C in the dark. Following incubation, the cells were washed three times with warm Tyrode’s solution to remove extracellular dye. Time-lapse Ca^2+^ imaging was performed using confocal laser scanning microscope (Zeiss LSM 800), through Zen (version 2.6, Zeiss) at 37°C. Fluo-4 was excited using a 488 nm laser, and emission was collected through a Plan-Neofluar 10×/0.30 objective. To accurately capture the rapid kinetic alterations and arrhythmic fluttering events in the mutant lines, high-speed 2D time-series acquisition was performed at a continuous scanning rate of 10 frames per second (fps) and for 243 frames per recording. Multiple regions of interest (ROIs) were recorded across independent cardiac sheets to ensure robust biological sampling. The data containing fluorescence intensity and time was exported as a CSV file to be operated on by CalDyn.

Intracellular cytosolic Ca^2+^ variations in hiPSC-NRs were measured in 60-day-old hiPSC-NRs enzymatically dissociated 7 days prior. The cells were loaded with 1 μM Fluo-4 AM in neurobasal medium for 15 min at 37°C. After incubation, the probe was removed and the medium replaced with Tyrode’s solution. Fluorescent changes were acquired using a ×63 objective (oil immersion), in frame-scan mode with an acquisition time of 5 to 10 min. The data were recorded in 16-bit format. Throughout the recording process, specific ROIs displaying fluorescence emissions were chosen. Upon completion of the recording, the background noise was subtracted, and the data was exported as a CSV file to be operated on by CalDyn.

Intracellular cytosolic Ca^2+^ variations in human primary skeletal myotubes were measured in response to electrical stimulations. Briefly, human primary skeletal myoblasts were plated on collagen-coated MatTek glass bottom 35-mm dishes (MatTek, P35GCOL-1.5-10-C). After five days in the proliferation medium, cell differentiation was induced and the cells were left for three days in the differentiation medium. The medium was replaced with Tyrode’s solution, and the cells were loaded with 2 μM Fluo-4 AM and in 1 μg/mL Hoechst 33342 for 20 min at 37°C. After incubation, the probe was removed, and the recordings were performed in Tyrode’s solution. Fluorescent changes were acquired using a ×40 objective (water immersion), with an acquisition time of 50 s. Acquisition parameters were set up to obtain a time per frame of roughly 110 ms (9 Hz). The differentiated myotubes were electrically stimulated using plunging electrodes from a Grass S88 stimulator with an amplitude of 15 V, a duration of 5 ms, and a frequency of 0.1 Hz. Each recording session began with a 10-s period with no stimulation, to detect spontaneous Ca^2+^ transients and set the signal baseline. This was followed by 40 s of electrical stimulation at 0.1 Hz. The data were exported as .CZI files, and image analysis was performed using FIJI software, where ROIs showing fluorescence emissions in different myotubes (3–6 myotubes per field) were selected. CalDyn adapts to the imaging modality: For line-scan data, amplitudes are reported in arbitrary units (AU) due to the absence of baseline recording in this high-speed acquisition mode. For frame-scan or whole-cell recordings, users may opt for baseline correction or F/F₀ normalization via the GUI. The resulting data was exported as a CSV file to be operated on by CalDyn.

### Immunofluorescence

The dissociated cells were initially fixed with 4% paraformaldehyde (PFA) for 15 min at room temperature, followed by three washes in 1× Phosphate Buffered Saline (PBS). After fixation, the cells underwent permeabilization using 0.1% Triton X-100 for 10 min at room temperature, and then were blocked with 0.1% Bovine Serum Albumin (BSA). Subsequently, the cells were incubated with the primary antibodies as outlined in Table 1 for 2 h at room temperature. This was followed by three additional washes before incubating the cells with the secondary antibodies, along with 0.1 μg/mL of 4′,6-diamidino-2-phenylindole (DAPI) for 1 h at room temperature. Finally, the cells were stored at 4°C, shielded from light. Immunofluorescence signals were visualized using an inverted fluorescence confocal microscope (Zeiss LSM 800), using ×20, ×40 and/or ×63 magnification.

**Table 1:** Primary antibodies used for immunofluorescence including the targeted cells and commercial references.

**Primary antibodies**	**Targeted cells**	**Commercial references**
β3-tubulin	Neurons	Sigma (#T8660)
Cardiac Troponin I	Cardiomyocytes	HyTest (#4 T21)
Alpha-actinin	Cardiomyocytes	Sigma (#A7811)
Alpha-actinin	Skeletal myotubes	Sigma (#A7811)

### Patch clamp recordings

Patch clamp experiments were conducted in isolated hiPSC-CMs at room temperature using an Axopatch 200B amplifier (Axon Instruments) as previously done [13]. The experiments were carried out at least seven days after dissociation of hiPSC-CMs at day 53 using TrypLE Express. AP was assessed using the whole-cell configuration in current clamp mode. AP were elicited at 1 Hz, following injection of 3 ms, 20–1500 pA rectangular current pulse. Borosilicate glass capillaries were used to make pipettes, which were subsequently fire-polished to obtain resistance between 2 and 3 MOhm. The intracellular pipette solution was composed (in mM): 10 NaCl, 122 KCl, 1 MgCl2, 1 EGTA, and 10 Hepes. The pH was adjusted to 7.3 with KOH. The bath solution was composed of (in mM): 154 NaCl, 5.6 KCl, 2 CaCl2, 1 MgCl2, 8 Glucose, and 10 Hepes. The pH was adjusted to 7.3 with 1 N NaOH. The data were recorded in an.ABF file, then the AP traces were exported into an Excel file for the analysis with CalDyn.

### Langendorff preparation

Hearts from 350 to 450 g Dunkin-Hartley guinea pigs were removed under isoflurane anesthesia (4% induction; 2% maintenance) and cannulated via Langendorff perfusion as previously described [14, 15]. Briefly, Tyrode’s solution containing (in mM) 118.0 NaCl; 4.7 KCl; 2.5 CaCl2; 25 NaHCO3; 1.2 KH2PO4; 1.2 MgSO4; 10.0 glucose, pH 7.4, and bubbled with 95% O2–5% CO2. To suppress motion artifacts, the heart was loaded with myosin uncoupler (−)-Blebbistatin (Sigma-Aldrich,), 5 μM. The heart was stained with a voltage-sensitive dye RH237 (Biotium, Fremont), 40 μM, and Ca^2+^ indicator Rhod-2 AM (Biotium), 10 μM.

### Optical mapping recordings

The optical mapping system consisted of two MiCAM N256 CMOS cameras (SciMedia Ltd., Costa Mesa, CA) connected to a MiCAM03 processing unit (SciMedia). The lens configuration provided a 16.5 mm × 16.5 mm field of view mapped onto the N256’s 17.6 mm × 17.6 mm sensor to create images with 256 × 256 resolution. Excitation illumination was provided by the LEX2-LZ4-G (SciMedia) green LED system connected to a COP1-A condensing lens (ThorLabs, Newton, NJ) via an LG-AD light guide. Excitation wavelengths pass through a 50 mm 520/35 nm filter (Semrock) and reflect off a 560 nm dichroic mirror (Semrock) toward the sample. Emissions from both dyes pass through the initial dichroic mirror and are then separated by an additional 662 nm dichroic mirror (Semrock) where the voltage fluorescence transmits, and the Ca^2+^ fluorescence is reflected. The voltage and Ca^2+^ fluorescence signals then pass through a final round of 700 nm long pass and 575/15 emission filters (Semrock), respectively. Data was collected (500 Hz sampling frequency) and analyzed in BV Workbench (SciMedia) and CalDyn.

### Statistical analysis

Statistical analyses were performed using GraphPad Prism version 9 (GraphPad Software). For comparisons in which the same recording was analyzed using two programs, measurements were matched at the same analytical level. Peak-level measurements generated by PeakInspector were aggregated within each cell or recording before comparison with the corresponding cell- or recording-level CalDyn output. Paired data were analyzed using two-sided paired *t-*tests when the distribution of the paired differences was considered approximately Gaussian or using two-sided Wilcoxon matched-pairs signed-rank tests otherwise. Comparisons between two independent groups were performed using two-sided unpaired *t-*tests for approximately Gaussian data or two-sided Mann–Whitney tests otherwise.

Agreement between analytical methods was assessed using Bland–Altman analysis. Differences were calculated as CalDyn minus the comparator method and plotted against the mean of the measurements obtained using the two methods. The mean paired difference was reported as the bias, and the lower and upper 95% limits of agreement were calculated as the mean bias ±1.96 standard deviations of the paired differences. Where applicable, the bias, standard deviation of the paired differences, 95% confidence interval of the mean paired difference, lower and upper 95% limits of agreement, and exact *p* values are reported. A non-significant paired comparison was not interpreted as evidence of equivalence or interchangeability between methods.

Detection performance was assessed against expert-reference annotations using sensitivity, precision, and F1 score. Specificity was not calculated because the number of true-negative opportunities in continuous time-series data depends on the arbitrary segmentation of non-event intervals. For each correctly matched event, onset-, peak-, and offset-timing errors were calculated as absolute errors and expressed as percentages of the local inter-event interval. Amplitude error was expressed as the absolute percentage difference relative to the expert-reference amplitude. Timing and amplitude errors are reported as median absolute errors because their distributions were not assumed to be Gaussian.

Synthetic-signal analyses were performed using 20 independent repetitions per condition. Detection performance was summarized using the percentage of correctly detected events and the median absolute timing and amplitude errors across repetitions. Noise level, sampling rate, baseline drift, and inter-event spacing were varied independently while the remaining simulation parameters were held constant. Parameter-sensitivity analyses were performed by varying the Savitzky–Golay filtering window, LOESS smoothing span, and derivative-threshold proportion individually from 0.5- to 2-fold of their modality-specific default values while holding the other parameters constant. Sensitivity, precision, and F1 score were recalculated against the same set of 142 expert-annotated events for each parameter setting.

Unless otherwise specified, descriptive values are reported as mean ± standard deviation. Individual paired observations are displayed in the method-comparison figures. The software-comparison, synthetic-signal, parameter-sensitivity, and biological-application outcomes were treated as exploratory because no single confirmatory primary endpoint was prespecified. Accordingly, exact two-sided unadjusted *P* values are reported without correction for multiple comparisons and are interpreted together with the observed effect sizes, agreement estimates, and biological-replication structure. Statistical significance was defined as *P* < .05. Where significance symbols are used, ^*^ indicates *P <* .05, ^**^ indicates *P <* .01, ^***^ indicates *P <* .001, and ^****^ indicates *P <* .0001.

### CalDyn usage

To facilitate user navigation, Fig. 1 illustrates the streamlined workflow of CalDyn. The process begins by launching the GUI (via MATLAB or the standalone Windows executable, Caldyn.exe). Users first set the data folder path and select the file(s) for analysis. CalDyn automatically detects peaks (e.g. Ca^2+^ transients or APs) using adaptive thresholding. If needed, users may manually refine peak detection (e.g. removing false positives). Next, baseline correction and peak classification options are applied to tailor the analysis to specific experimental conditions. Finally, results are saved in an Excel-compatible spread sheet format for further statistical evaluation. The standalone Windows version, compiled using MATLAB Runtime, ensures accessibility for users without a full MATLAB license.

- Organization of the data

**Figure 1:**
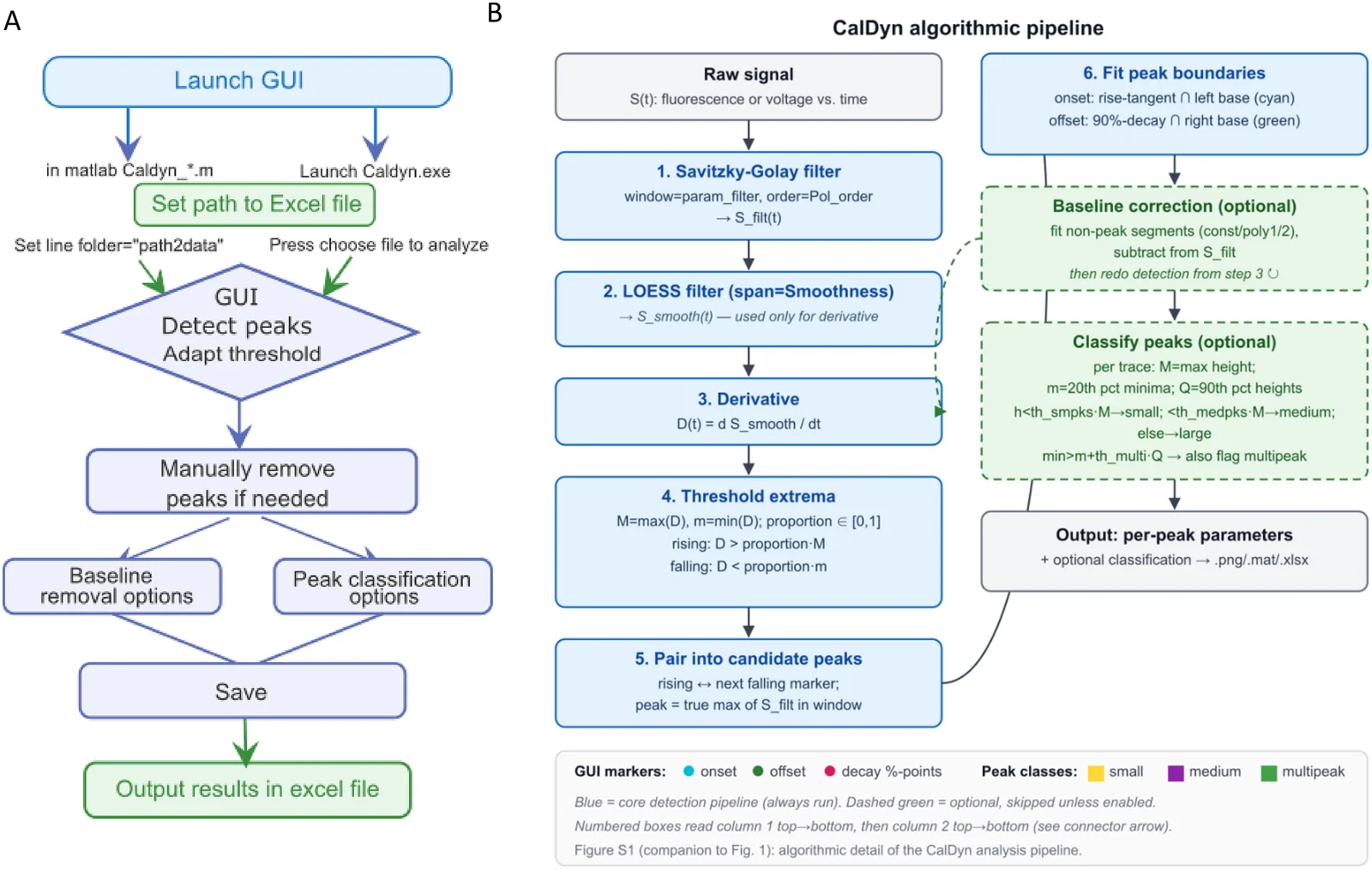
**A** CalDyn workflow diagram. The process begins with GUI launch, followed by file selection, automated peak detection, manual refinement, parameter customization, and result exportation. **B:** CalDyn algorithmic pipeline. Blue boxes show the core detection pipeline (always run): Savitzky–Golay filtering, LOESS filtering (for the derivative only), derivative computation, threshold-based extrema detection, pairing into candidate peaks, and boundary fitting. Dashed green boxes show the two optional branches (baseline correction, peak classification), skipped unless enabled by the user. See the CalDyn Algorithm section for full notation.

The software accepts both Excel and csv files, the first column will represent the time vector, and the following columns will be the different electrical, fluorescent measurements at different locations, or on different cells.

All the parameters calculated afterwards will use the units which are used in the data. Conversion, if necessary, could be done afterwards.

- Quick description of the GUI (details on GitHub)

In the GUI appear two graphs (Fig. 2):

- On the left, the filtered signal is shown in blue, while the original signal of intracellular Ca^2+^ dynamics in hiPSC-NRs is displayed in light gray. When the filtering is minimal, the original signal may be obscured and hidden by the blue lines.
- On the right is the filtered derivative of the filtered signal in red, superimposed with the signal itself. A peak in the original signal, will transform into a maximum and a minimum in the derivative signal. The two dashed lines represent the two thresholds for the detection of the ascending part of the peak and of the descending one. This enables us to visualize which peaks are detected.
- Quick Start Guide:

1. Open MATLAB
2. Open the script CalDyn_Ca
3. “Change folder” and choose the data file
4. Run the script
5. A window opens

**Figure 2:**
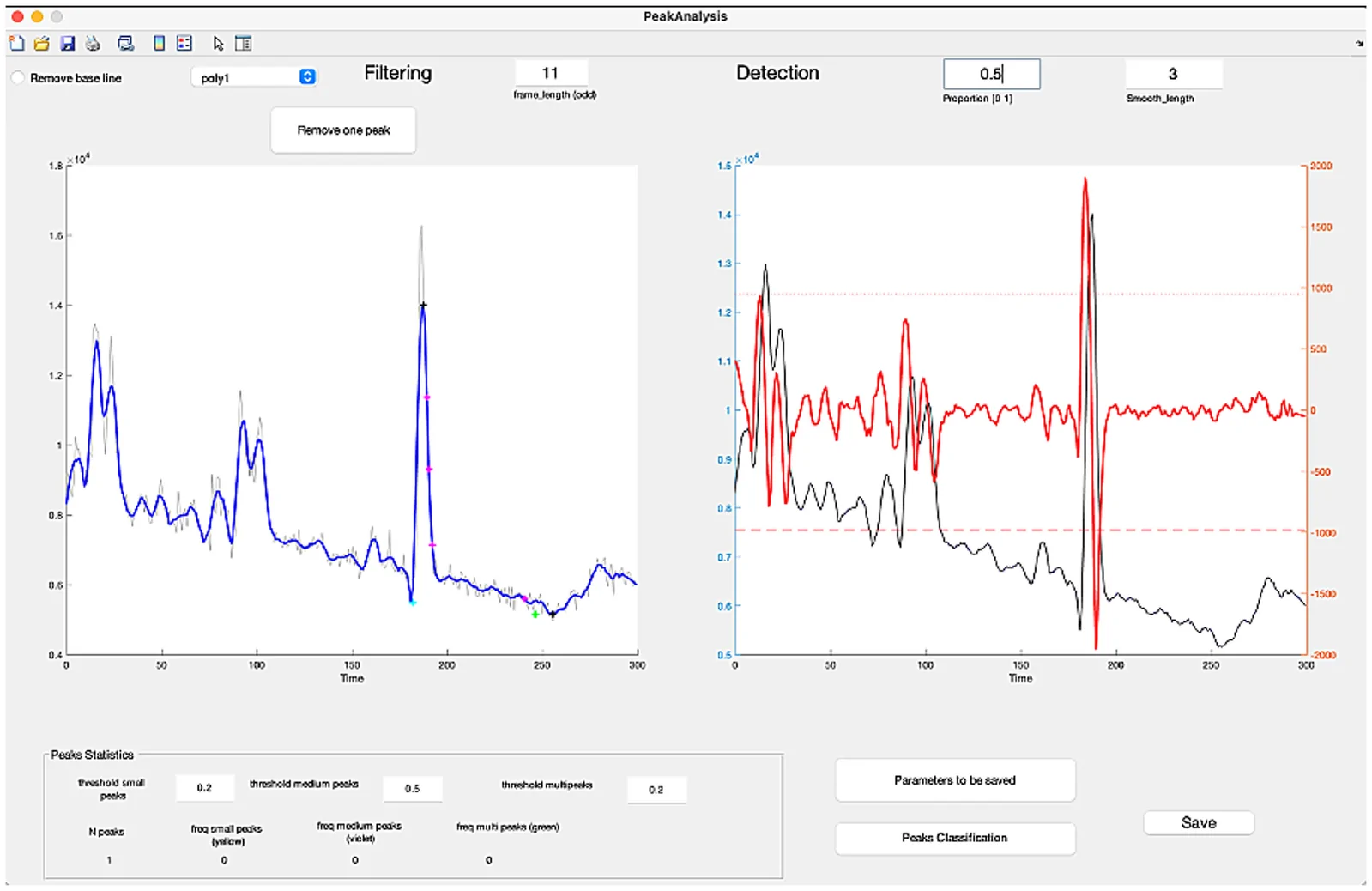
Example of intracellular Ca^2+^ signal in hiPSC-NRs as presented in the GUI CalDyn interface.

For example:

1. Vary the following parameters, at the user’s discretion
  - Filtering
  - Detection (proportion: baseline and smooth derivative)
2. Select the parameters that you want to be saved, using the parameter window. You can choose to save the mean and/or the median and/or the standard deviation of all the checked parameters.
3. When finished, press save.
4. The following position automatically opens
5. Repeat the same procedure

### CalDyn algorithm

A schematic of the full algorithmic pipeline described in this section, including the two optional branches (baseline correction and peak classification), is provided in Fig. 1B.

- Algorithm for peak detection:

There are many algorithms for local maxima detection, each with its own set of input parameters such as minimum peak height, peak prominence, threshold, and peak distance. However, we chose not to pursue this approach for two reasons. Firstly, the signals we analyze can vary rapidly over time, making it challenging to adjust the parameters for each recording. This is especially true for signals with a fluctuating baseline. To achieve more robust detection, we decided to use the derivative of the signals, which produces a series of maxima and minima for each peak. We thus search for a maximum in the original signal, only in between a significant maximum and minimum of the derivative.

- Data Filtering

Data is filtered in two steps:

For each cell, the initial signal (fluorescence or voltage as a function of time) is subjected to a Savitzky–Golay filtering procedure [16]. This filtering step serves to smooth the signal and eliminate noise. The user is given the flexibility to select the frame length for the filtering process. Users must search for a balance between achieving accuracy, by avoiding aliasing, and maintaining smoothness in the fitting. This can be achieved by comparing the light grey (original signal) and blue (filtered signal).

The polynomial frame length represents the number of data points considered within the filtering window at any given time. The optimal frame length for Savitzky–Golay filtering, based on the chosen order, can be determined using the guidelines provided by Sadeghi and Behnia [17].

Before taking the derivative, the signal is filtered a second time using the “LOESS” scheme algorithm with the “smooth function” of MATLAB [18]. This second filtering is for helping to only select the proper peaks of interest and to get rid of the rest of the signal. This LOESS-smoothed signal is used only to locate the candidate maxima/minima of the derivative (see below); peak amplitude, onset, and offset are all measured on the once-filtered (Savitzky–Golay) signal, which is the mechanistic reason this second filtering step does not bias peak-position accuracy (no aliasing problem for this second filtering step). We validated this directly: across 142 manually annotated peaks spanning all cell/tissue types reported in this manuscript (see the new expert reference validation section below), median peak-position error was ≈0%.

Let *M* = max(*D*) and *m* = min(*D*) denote the absolute maximum and minimum of the derivative *D* of the LOESS-smoothed signal. Local maxima of *D* above *proportion* × *M* (a user-defined threshold between 0 and 1, interactively adjustable in the GUI, dashed line in GUI panel B) and local minima of *D* below *proportion* × *m* (note *m* < 0, so this threshold is itself negative, no sign inversion is applied) are retained as candidate rising and falling markers, respectively. Once these maxima and minima are calculated, they are associated by pairs to define a peak, whose properties are further calculated.

- Finding the base of the peaks:

Although finding peaks is usually rather straightforward, defining the starting and ending point of the peak can prove to be difficult, especially if noise is important.

Here, we chose to utilize the inherent dissymmetry of the Ca^2+^ peaks, which show a faster rise and a slower descent, so that both sides are treated slightly differently. This asymmetric-kinetics assumption is well suited to Ca^2+^ transients and, as shown in the Results, transfers well to cardiac action potentials and optical-mapping voltage signals, which share the same fast-upstroke/slow-repolarization shape. We have not validated it on signal types with symmetric or reversed kinetics (e.g. certain neuronal Ca^2+^ events), and we do not claim applicability beyond waveforms sharing this asymmetry.

For the rise, we calculate the position and the maximum of the slope, we then fit the rising part of the peak with a linear curve of that slope and calculate the time value corresponding to its intersection with a constant line whose value is the minimum of the signal on the left of the peak (or zero if the baseline reference option has been chosen) (Fig. 2). The intersection point will be plotted in cyan in the GUI.

For the descent, we calculate the time it takes for the signal to go back to 90% of its height and calculate the average slope of the signal between the time of the maximum and that point; we again fit the signal with a linear curve of that slope and find its intersection with a constant line whose value is the minimum of the signal on the right of the peak (or zero if the baseline reference option is chosen) (Fig. 2). The intersection point is plotted in green in the GUI.

Manual peak removal, when used, is strictly subtractive: it lets the user delete a false-positive peak after automated detection, but never adds a peak and never alters the boundary/amplitude calculations of the peaks that remain. Automated detection and manual curation are therefore fully separable in the saved output.

- Baseline detection:

If needed, a baseline detection scheme can be implemented. A first peak detection is made, then the part of the signal which is not associated with peaks will be fitted either by a constant or a polynomial of order 1 or 2, which will define the baseline. The baseline is then subtracted to the original signal, and a new peak detection path is performed. If the baseline reference option has been chosen, this means that all minima will be put to zero.

- Peak classification: Detection of different types of peaks

Once all peaks are detected they can be classified if the peak classification option was chosen. In this case, the user defines a threshold for small peaks (*th_smpks*), one for medium peaks (*th_medpks*), and one for multipeaks (*th_multi*).

We calculate three quantities for assigning peaks to their categories, recalculated independently for every analyzed trace (see the Discussion for the implications of this choice for between-group comparisons):

- *m*: the 20th percentile of all the minima associated with peaks in that trace (previously referred to imprecisely as the “second quantile”).
- *Q*: the 90th percentile of all peak heights in that trace (previously the “ninth quantile”).
- *M*: the maximum peak height in that trace.

Let h_left and h_right denote the height of a given peak measured from its left and right minimum, respectively, and h = max(h_left, h_right). A peak is classified as follows:

- small if h < *th_smpks* × M;
- medium if *th_smpks* × M ≤ h < *th_medpks* × M;
- large otherwise;
- and, independently of the above, also flagged as a multipeak if its left or right minimum exceeds *m* + *th_multi* × Q (i.e. the signal does not return to baseline on that side before the next peak begins). Because of noise, this comparison is itself threshold-based (*th_multi*) rather than a strict equality to baseline.

For visualization purposes in the GUI, small peaks will be drawn in yellow, medium ones in purple and multipeaks in green.

Peak classification is a deterministic labeling rule, not an estimate: given a peak’s already-measured height and the three threshold values above, the resulting category follows from a single arithmetic comparison with no ambiguity. The default threshold values (0.2/0.5/0.2) and the resulting categories carry no inherent biological meaning of their own; they are generic, user-adjustable analytical settings, and it is the responsibility of the user to choose thresholds appropriate to their own experimental question and to establish, for their own system, what a given category corresponds to biologically.

Default values and permitted ranges for all user-adjustable parameters described above are given in Table 2.

**Table 2:** Default settings and permitted ranges for CalDyn’s user-adjustable parameters.

**Parameter**	**Default**	**Meaning**	**Range**
param_filter	111	Savitzky–Golay window (points)	odd integer, > Pol_order
Pol_order	2	Savitzky–Golay polynomial order	typically 2–4
Smoothness	200	LOESS window (2nd filtering pass)	tuned per dataset
Cut_freq	20	FFT cutoff frequency (MEA signals only)	dataset-dependent
proportion	0.1	Derivative threshold fraction	0–1
th_smpks / th_medpks / th_multi	0.2 / 0.5 / 0.2	Peak-classification thresholds	0–1 each
baselinefit	off	Baseline fit: off / constant / poly. order 1 / poly. order 2	—
bool_baselineref	off	If on, all peak minima are referenced to 0	—

### CalDyn output

For each peak detected in the signal, the following parameters (gathered in Table 3 below) are systematically measured (for a detailed description, see the Readme.md file in the GitHub folder).

**Table 3:** CalDyn output.

Temporal parameters	+Ascending time + Decay time + Time for the signal to decay of 95%, 90%, 80%, 70%, 50%, 30%, 20% of its maximum amplitude+ time between the end of a peak and the beginning of the next one +T time in between two peaks+Corrected according to Bazett formula
Slope parameters	+Ascending slope + Average decays slope + Average decay slope during the first half of the decrease, Average decay slope in the second half of the decrease, standard deviation of the decay slope (these last three parameters are especially interesting when the peak shape is departing from a triangular shape)
Amplitude parameters	+Amplitude of the increase + Amplitude of the decrease (can be different from the previous parameter, if the signal is not returning completely back to its base value at the end of the peak) + Maxima and Minima values of the peaks, +Signal to noise ratio (the Signal is defined as the average value of the peak amplitude, and noise as the 90th centile of the base line minus its average)
Integral parameter	Area under the curve of each peak (AUC)
Classification parameters	Frequency of small, medium peaks and multipeaks (see the section devoted to peak classification)

The user can select the parameters that need to be output using the parameter window.

The output of the analysis is the following and is saved in a folder result in the same directory as the data:

1. A .png file of the GUI window for each signal analyzed. This enables, in the case of an automatic analysis, to rapidly check that the measurements were done properly
2. A .mat file where a MATLAB structure of all the results is saved
3. An excel file where the mean and/or the median and/or the standard deviation (and/or can again be chosen checking the corresponding boxes in the parameter window) of all the parameters are saved for each temporal signal in the initial excel file. The user can choose to get the results in column or in line changing the col_line parameter from 0 to 1. If the peak classification has been chosen, one will find in the excel file supplementary sheets, named “small_peaks,” “medium_peaks,” “large_peaks,” where all the statistics on the parameters chosen are calculated respectively only on the small/medium/large peaks.

## Results

Using MATLAB, we developed a script for analyzing Ca^2+^ dynamics in excitable cells. This user-friendly software, which launches a Graphical User Interface, is compatible with Windows, MacOS and Linux. We present here results on various cell types and signals, showing the capacities of the software. All the examples presented can be retrieved on GitHub for testing and demonstrating ease of use.

### Proof of concept

To thoroughly assess CalDyn, we tested it across three distinct differentiated cell populations: hiPSC-CMs, hiPSC-NRs and differentiated skeletal myotubes.

### Paired comparison of CalDyn and PeakInspector for Ca^2+^-transient analysis in healthy hiPSC-CMs

Cardiomyocytes are characterized by their sophisticated contractile systems, heavily influenced by specialized intracellular Ca^2+^ dynamics. To validate the outcomes produced by CalDyn, we utilized the previously verified PeakInspector script as reference [7, 8, 11, 19]. The obtained differentiated ventricular-like hiPSC-CMs are large cells exhibiting the typical sarcomeric pattern revealed by alpha-actinin and cardiac troponin I, 2 major proteins of the cardiac contractile apparatus revealed by immunofluorescence (Fig. 3A).

**Figure 3:**
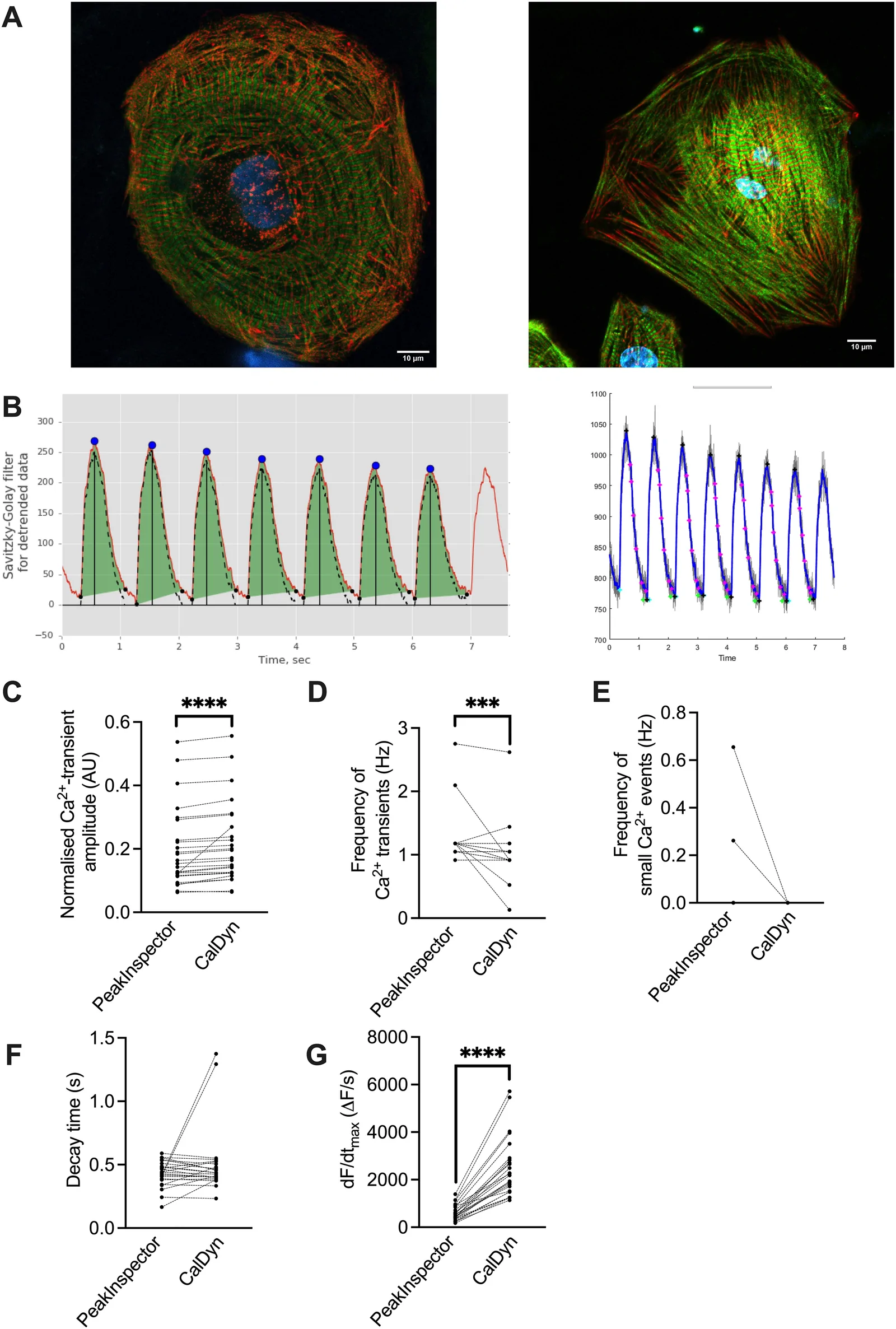
Paired comparison of intracellular Ca^2+^-transient parameters obtained using PeakInspector and CalDyn in healthy control hiPSC-CMs. (A) Immunofluorescence staining of cardiomyocytes showing □-actinin (red), cardiac troponin I (green), and DAPI (blue). The scale bar is 10 μm. (B) Representative recording illustrating cardiac Ca^2+^ transients obtained from PeakInspector and CalDyn. **(C-G)** Paired cell-level comparisons of normalized Ca^2+^-transient amplitude **(C)**, Ca^2+^-transient frequency **(D)**, small Ca^2+^-event frequency **(E)**, decay time **(F)**, and maximum Ca^2+^-transient rise rate, dF/dt_max_  **(G)**, obtained using PeakInspector and CalDyn. Peak-level measurements generated by PeakInspector were summarized within each cell to provide the same cell-level analytical unit as the corresponding CalDyn output. Each symbol represents one cell, and lines connect measurements obtained from the same recording using the two programs. Bland–Altman analyses of agreement between CalDyn and PeakInspector are shown in Supplementary Fig. 1 and summarized in Table 4.

We monitored the intracellular Ca^2+^ transients in hiPSC-CMs using fluorescent confocal microscopy. The resulting Ca^2+^ transients were processed with PeakInspector and CalDyn (Fig. 3B). Because PeakInspector reports measurements for individual peaks, whereas CalDyn summarizes the detected peaks within each cell, the PeakInspector peak-level measurements were first aggregated at the cell level. This provided the same analytical unit for both programs and enabled paired comparisons of 25 matched cells. The comparisons included normalized Ca^2+^-transient amplitude, principal Ca^2+^-transient frequency, small-event frequency, decay time, and maximum Ca^2+^-transient rise rate, dF/dt_max_ (Fig. 3C–G). CalDyn yielded a modest but consistently higher normalized Ca^2+^-transient amplitude than PeakInspector (0.2151 ± 0.1287 AU versus 0.1982 ± 0.1270 AU, respectively; Wilcoxon matched-pairs test, *P* = .0002; Fig. 3C and Table 4). Conversely, CalDyn reported a lower principal Ca^2+^-transient frequency than PeakInspector (1.0270 ± 0.4031 Hz versus 1.2310 ± 0.3781 Hz; *P =* .0002; Fig. 3D and Table 4). These differences likely reflect, at least in part, differences in event detection, classification, and waveform processing between the two programs. Small Ca^2+^ events were uncommon in this healthy-cell dataset. PeakInspector detected small events in a limited number of cells, whereas CalDyn did not classify any events as small under the applied settings. The paired comparison did not identify a statistically detectable difference in small-event frequency (*P =* .5000; Fig. 3E and Table 4). Decay-time estimates also did not show a statistically detectable paired difference between the programs, although CalDyn produced a numerically higher mean value than PeakInspector (0.5046 ± 0.2594 s versus 0.4293 ± 0.0996 s; *P =* .0903; Fig. 3F and Table 4). The absence of a significant paired difference should not be interpreted as demonstrating equivalence between the methods. The maximum Ca^2+^-transient rise rate differed substantially between the two programs. CalDyn produced higher dF/dt_max_ values than PeakInspector (2464 ± 1240 ΔF/s versus 603.2 ± 321.7 ΔF/s; *P <* .0001; Fig. 3G and Table 4). This difference probably reflects the distinct computational definitions used by the programs: PeakInspector estimates the rise rate from amplitude and rise time, whereas CalDyn calculates the slope from the ascending phase of the transient. Accordingly, dF/dt_max_ values obtained using the two approaches should not be regarded as directly interchangeable.

**Table 4:** Summary of Bland–Altman agreement between CalDyn and PeakInspector for the Ca^2+^ transient parameters in healthy control hiPSC-CMs. Peak-level measurements generated by PeakInspector were summarized at the cell level to permit comparison with the corresponding cell-level CalDyn outputs. Bias was calculated as the mean paired difference between CalDyn and PeakInspector measurements (CalDyn - PeakInspector). Accordingly, a negative bias indicates that CalDyn produced lower estimates than PeakInspector, whereas a positive bias indicates that CalDyn produced higher estimates. The standard deviation represents the dispersion of the paired differences between the two methods. The lower and upper 95% limits of agreement were calculated as the mean bias ±1.96 standard deviations of the paired differences. LoA, limits of agreement; dF/dt_max_, maximum Ca^2+^-transient rise rate.

**Parameter**	**Unit**	**Matched cells, *n***	**PeakInspector, mean ± SD**	**CalDyn, mean ± SD**	**Mean bias: CalDyn - PeakInspector**	**SD of paired differences**	**Lower 95% LoA**	**Upper 95% LoA**	**Paired test**	***P*-value**
Normalized amplitude	Dimensionless^*^	25	0.1982 ± 0.1270	0.2151 ± 0.1287	0.0169	0.028	−0.0380	0.0717	Wilcoxon matched-pairs test	.0002
Ca^2+^-transient frequency	Hz	25	1.2310 ± 0.3781	1.0270 ± 0.4031	−0.2043	0.3165	−0.8247	0.4161	Wilcoxon matched-pairs test	.0002
Small-event frequency	Hz	25	0.0367 ± 0.1390	0.0000 ± 0.0000	−0.0367	0.139	−0.3092	0.2358	Wilcoxon matched-pairs test	.5
Decay time	s	25	0.4293 ± 0.0996	0.5046 ± 0.2594	0.0753	0.2649	−0.4440	0.5945	Wilcoxon matched-pairs test	.0903
Maximum rise rate, dF/dt_max_	ΔF/s	25	603.2 ± 321.7	2464 ± 1240	1861	981.2	−62.1	3784	Wilcoxon matched-pairs test	<.0001

Bland–Altman analysis was used to further quantify systematic bias and recording-level agreement between CalDyn and PeakInspector (Supplementary Fig. 1 and Table 4). The mean CalDyn-minus-PeakInspector biases were 0.0169 for normalized amplitude, −0.2043 Hz for Ca^2+^-transient frequency, −0.0367 Hz for small-event frequency, 0.0753 s for decay time, and 1861 ΔF/s for dF/dt_max_. The corresponding 95% limits of agreement varied among parameters, indicating that a relatively small mean bias for some outcomes could coexist with appreciable disagreement for individual cells. In particular, the limits of agreement were relatively broad for transient frequency, decay time, and dF/dt_max_. Collectively, these analyses demonstrate that CalDyn can detect and quantify Ca^2+^ transients in healthy hiPSC-CMs while also identifying systematic differences attributable to the analytical definitions and processing procedures implemented by each program.

### CalDyn detects complex intracellular Ca^2+^-transient patterns in arrhythmic hiPSC-CMs

We next evaluated CalDyn using intracellular Ca^2+^-transient recordings from arrhythmic hiPSC-CMs carrying a pathogenic RyR2 variant associated with polymorphic ventricular tachycardia at rest (PMVT), as previously described [9]. The same 17 recordings were analyzed using CalDyn and PeakInspector, enabling paired comparisons between the two programs. PeakInspector peak-level measurements were summarized within each recording to provide the same analytical unit as the corresponding CalDyn output.

Both programs identified the principal Ca^2+^ transients and heterogeneous waveform features present in the PMVT recordings, including candidate low-amplitude diastolic events and candidate multicomponent transients (Fig. 4A). CalDyn produced lower normalized Ca^2+^-transient amplitude estimates than PeakInspector (0.671 ± 0.472 AU versus 1.853 ± 1.302 AU, respectively; Fig. 4B and Table 5). Conversely, CalDyn detected a higher frequency of principal Ca^2+^ transients than PeakInspector (1.805 ± 1.134 Hz versus 0.666 ± 0.308 Hz; Fig. 4C and Table 5). The mean frequency of candidate low-amplitude diastolic events was numerically higher with CalDyn than with PeakInspector (0.586 ± 1.198 Hz versus 0.322 ± 0.323 Hz), but this difference was strongly influenced by one recording with a CalDyn estimate exceeding 5 Hz and did not show a consistent direction across the matched observations (Fig. 4D and Table 5). In contrast, CalDyn identified a higher frequency of candidate multicomponent Ca^2+^ transients than PeakInspector (1.076 ± 0.894 Hz versus 0.269 ± 0.264 Hz; Fig. 4E and Table 5). Because these event classes are determined using method-specific detection and classification procedures, the observed differences represent differences in software detection behavior rather than independent evidence that one method is more accurate. CalDyn also produced shorter decay-time estimates than PeakInspector (0.376 ± 0.196 s versus 0.606 ± 0.265 s; Fig. 4F and Table 5). In contrast, the mean maximum Ca^2+^-transient rise rates were comparable at the group level—83,829 ± 93,419 ΔF/s with CalDyn and 82,400 ± 79,829 ΔF/s with PeakInspector, but the paired observations showed considerable recording-level variability (Fig. 4G and Table 5). Because PeakInspector derives this parameter from transient amplitude and rise time, whereas CalDyn calculates the slope of the ascending phase, the resulting dF/dt_max_ values should not be assumed to be directly interchangeable. The low-amplitude and multicomponent labels describe waveform classes assigned by each analytical method and do not independently confirm a specific Ca^2+^-release mechanism.

**Figure 4:**
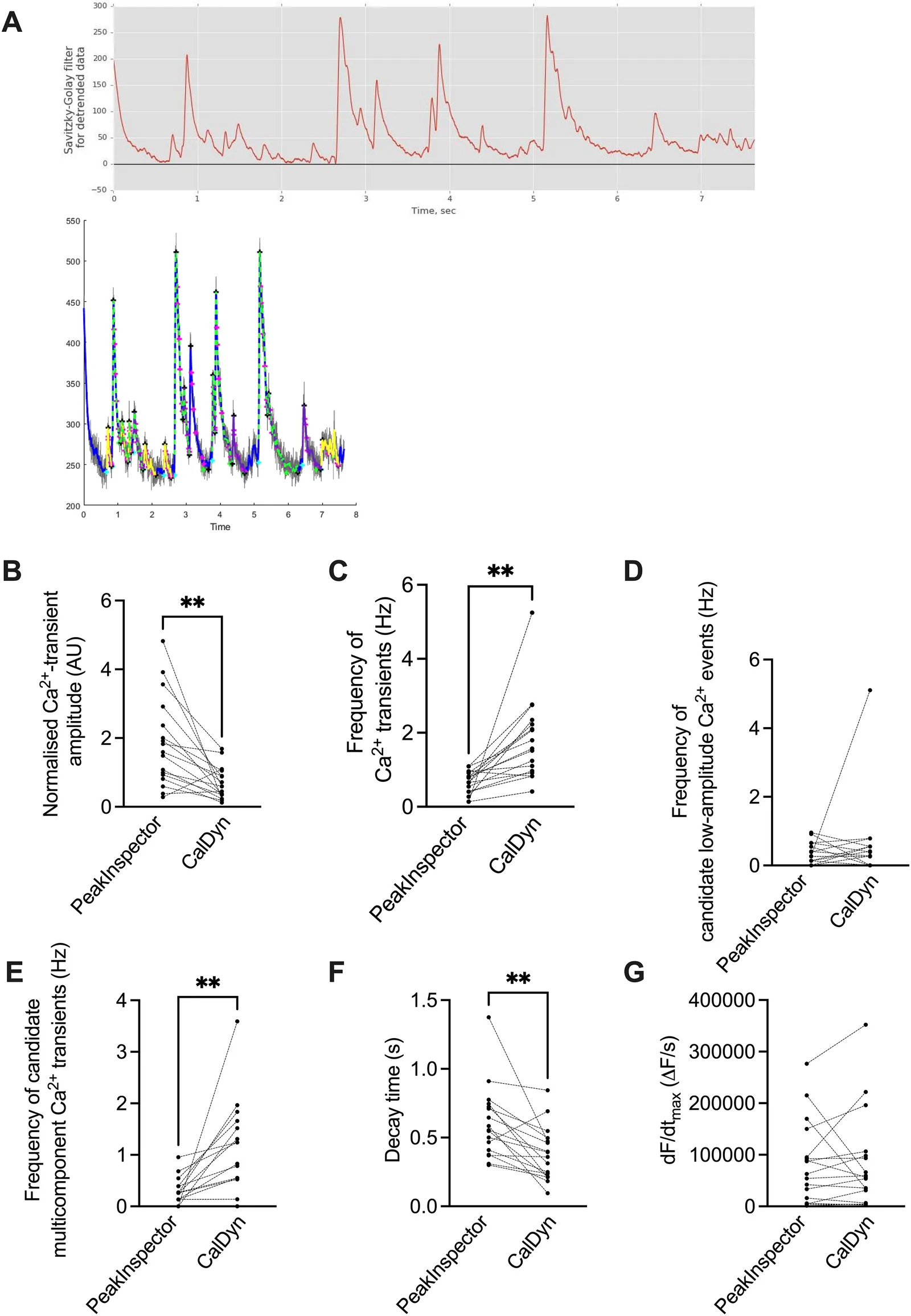
Paired comparison of intracellular Ca^2+^-transient parameters obtained using PeakInspector and CalDyn in arrhythmic PMVT hiPSC-CMs. (A) Representative recording exhibiting complex intracellular Ca^2+^ activity analyzed using PeakInspector (top) and CalDyn (bottom), including principal Ca^2+^ transients, candidate low-amplitude diastolic events, and candidate multicomponent Ca^2+^ transients. The PeakInspector output shows the filtered and detrended signal, whereas the CalDyn output shows the original and filtered signals together with the automatically identified peak maxima, waveform-analysis points, and event classifications. (B-G) Paired recording-level comparisons of normalized Ca^2+^-transient amplitude (B), principal Ca^2+^-transient frequency (C), candidate low-amplitude diastolic-event frequency (D), candidate multicomponent Ca^2+^-transient frequency (E), decay time (F), and maximum Ca^2+^-transient rise rate, dF/dt_max_ (G). Peak-level measurements generated by PeakInspector were summarized within each recording to provide the same analytical unit as the corresponding CalDyn output. Each symbol represents one recording, and lines connect measurements obtained from the same recording using the two programs. Exact p values and paired effect estimates are reported in Table 5. Bland–Altman analyses are shown in Supplementary Fig. 2. dF/dt_max_, maximum Ca^2+^-transient rise rate; PMVT, polymorphic ventricular tachycardia at rest. The candidate-event labels describe waveform characteristics assigned by the analytical methods and do not constitute independent biological confirmation of the underlying Ca^2+^-release mechanism.

**Table 5:** Paired comparison and Bland–Altman agreement between CalDyn and PeakInspector for intracellular Ca^2+^-transient parameters in arrhythmic PMVT hiPSC-CMs. Peak-level measurements generated by PeakInspector were summarized within each recording to provide the same analytical unit as the corresponding CalDyn output. Descriptive values are reported as mean ± SD. Bland–Altman bias was calculated as the mean paired difference between CalDyn and PeakInspector measurements (CalDyn - PeakInspector). Accordingly, a negative bias indicates lower estimates obtained using CalDyn, whereas a positive bias indicates higher estimates obtained using CalDyn. The standard deviation of the paired differences describes the dispersion of the individual differences between the two programs. The lower and upper 95% limits of agreement were calculated as the mean bias ±1.96 standard deviations of the paired differences. LoA, limits of agreement; PMVT, polymorphic ventricular tachycardia at rest; dF/dt_max_, maximum Ca^2+^-transient rise rate.

**Parameter**	**Unit**	**Matched recordings, n**	**PeakInspector, mean ± SD**	**CalDyn, mean ± SD**	**Mean bias: CalDyn - PeakInspector**	**SD of paired differences**	**Lower 95% LoA**	**Upper 95% LoA**	**Paired test**	***P*-value**
Normalized Ca^2+^-transient amplitude	Dimensionless^*^	17	1.853 ± 1.302	0.671 ± 0.472	−1.182	1.294	−3.718	1.354	Paired *t*-test	.0017
Principal Ca^2+^-transient frequency	Hz	17	0.666 ± 0.308	1.805 ± 1.134	1.139	1.217	−1.246	3.525	Paired *t*-test	.0014
Candidate low-amplitude diastolic-event frequency	Hz	17	0.322 ± 0.323	0.586 ± 1.198	0.264	1.235	−2.158	2.685	Wilcoxon matched-pairs signed rank test	.7148
Candidate multicomponent Ca^2+^-transient frequency	Hz	17	0.269 ± 0.264	1.076 ± 0.894	0.807	0.900	−0.957	2.571	Paired *t*-test	.0020
Decay time	s	17	0.606 ± 0.265	0.376 ± 0.196	−0.231	0.252	−0.725	0.264	Paired *t*-test	.0017
Maximum rise rate, dF/dt_max_	ΔF/s	17	82,400 ± 79,829	83,829 ± 93,419	1429	65,240	−126,442	129,300	Wilcoxon matched-pairs signed rank test	.4586

Bland–Altman analyses were used to quantify the systematic bias and recording-level agreement between the programs (Supplementary Fig. 2 and Table 5). The mean CalDyn-minus-PeakInspector biases were −1.182 for normalized amplitude, 1.139 Hz for principal Ca^2+^-transient frequency, 0.264 Hz for candidate low-amplitude event frequency, 0.807 Hz for candidate multicomponent transient frequency, −0.231 s for decay time, and 1429 ΔF/s for dF/dt_max_. The 95% limits of agreement were broad for several outcomes, particularly the event-frequency and dF/dt_max_ measurements, showing that limited average bias for an outcome can coexist with substantial disagreement for individual recordings.

Collectively, these analyses demonstrate that CalDyn can detect and quantify complex Ca^2+^-transient patterns in arrhythmic PMVT hiPSC-CMs. They also identify systematic differences between CalDyn and PeakInspector that likely arise from differences in peak detection, event classification, waveform-boundary definitions, and parameter calculation. The two programs should therefore not be considered interchangeable for all outcomes without method-specific interpretation and visual quality control.

### CalDyn detects Ca^2+^-transient alterations in genetically and pharmacologically perturbed 2D patient-derived cardiac sheets

To demonstrate the application of CalDyn to a multicellular genetic disease model, intracellular Ca^2+^ transients were analyzed in cardiac sheets generated from the LMNA p.H222P hiPSC line and its gene-corrected isogenic control. This patient-derived model has recently been used to investigate contractile dysfunction and altered Ca^2+^ handling associated with LMNA-related cardiac laminopathy [10]. In these multicellular preparations, Ca^2+^ signals reflect the integrated activity of interconnected, predominantly ventricular-like hiPSC-CMs [9]. CalDyn extracted the inter-transient period, multipeak-event frequency, rise time, and decay time from multiple ROIs within each sheet (Supplementary Fig. 3 and movies ISO_S1 and LMNA_S2). The ROI-level distributions showed descriptive differences between the LMNA p.H222P and isogenic-control cardiac sheets. Because the dataset comprised four independent cardiac sheets, with multiple ROIs nested within each sheet, no inferential statistical comparison was performed. These results are presented as an application example rather than as a population-level genotype comparison.

CalDyn was subsequently applied to Ca^2+^-transient recordings from healthy control cardiac sheets exposed acutely to vehicle or 5 nM Pacific ciguatoxin-1B (CTX-1B). CTX-1B modulates voltage-gated sodium-channel activity and is one of the principal toxins associated with ciguatera fish poisoning [20]. Representative recordings showed more closely spaced and lower-amplitude Ca^2+^ transients following CTX-1B exposure (Supplementary Fig. 4A). CalDyn extracted Ca^2+^-transient amplitude, inter-transient period, rise time, and decay time from multiple ROIs within each sheet (Supplementary Fig. 4B–E). The ROI-level distributions illustrate the changes observed in the analyzed sheets following CTX-1B exposure. Because the dataset comprised four independently treated cardiac sheets, with multiple ROIs nested within each sheet, no inferential statistical comparison was performed. The experiment is therefore presented as a descriptive pharmacological application of CalDyn.

### CalDyn is able to monitor intracellular Ca^2+^ handling under electrical pacing in primary human skeletal myotubes

Following the analysis of spontaneously beating hiPSC-CMs, we next aimed to verify the compatibility of CalDyn for analyzing the intracellular Ca^2+^ properties in other myocytes that do not beat spontaneously and therefore require electrical stimulation to release Ca^2+^ and allow excitation-contraction coupling (ECC). We performed confocal fluorescent microscopy on differentiated primary human healthy skeletal myotubes exhibiting sarcomeric organization (Fig. 5A). Following filtering, our results indicated that CalDyn detects four well-defined pace-inducing Ca^2+^ transients with an amplitude over tenfold compared to the mean noise amplitude (Fig. 5B and C).

**Figure 5:**
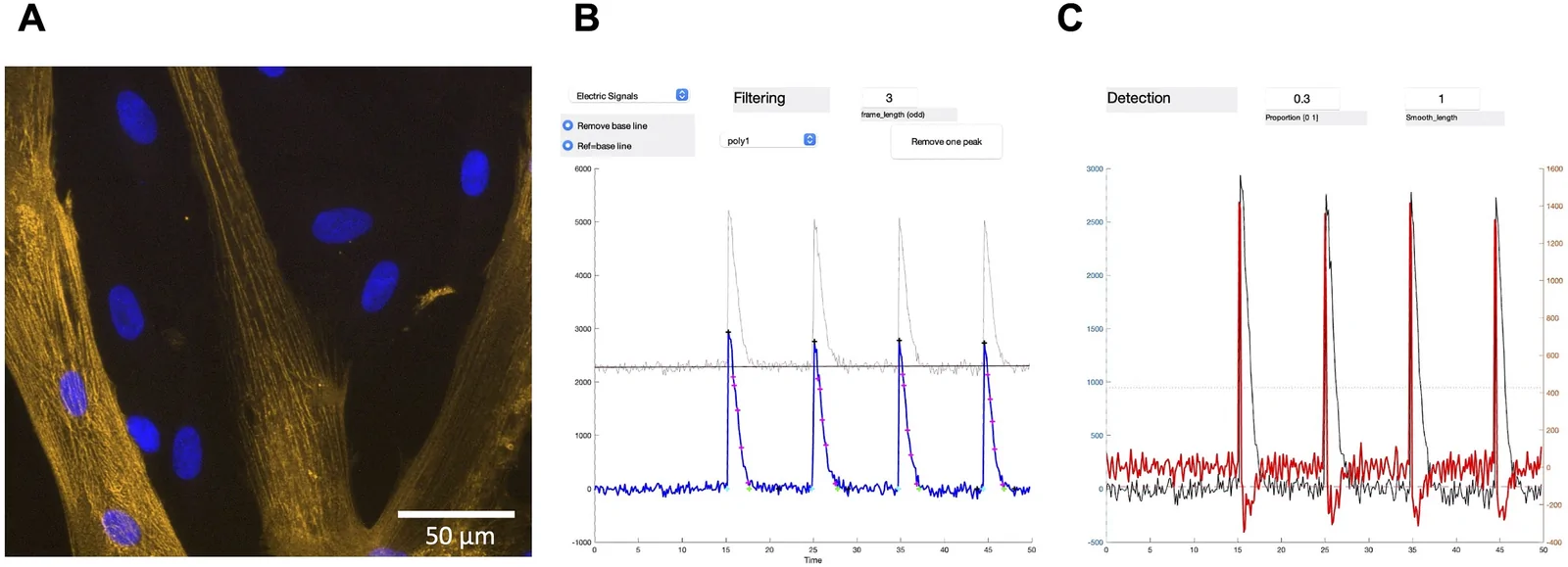
Intracellular Ca^2+^ dynamics in primary healthy human myotubes detected by CalDyn. (A) Immunofluorescence imaging of primary healthy human myotubes, labeled with ⍺-actinin (orange) and nuclear DNA stained with DAPI (blue). (B and C) Typical plot showing the Ca^2+^ response of a myotube to an electrical stimulus, obtained using CalDyn.

### CalDyn is able to sense unstable intracellular Ca^2+^ handling in hiPSC-NRs

The analysis of intracellular Ca^2+^ variations in neurons is a complex task, chiefly due to the slow, variable amplitude and diverse Ca^2+^ kinetic profiles that are predominantly linked to the Ca^2+^ release from RyR and IP3R [21, 22]. Given that the intricacies of Ca^2+^ dynamics are not singular in occurrence, there is a significant need to establish a computational tool designed to facilitate the comprehension, investigation, and analysis of these dynamics. Using confocal fluorescent microscopy on differentiated forebrain hiPSC-derived NRs of high cellular density (Fig. 6A), we observed highly heterogeneous intracellular Ca^2+^ release events. After applying filters, CalDyn identifies consistent primary Ca^2+^ release peaks (Fig. 6B and C).

**Figure 6:**
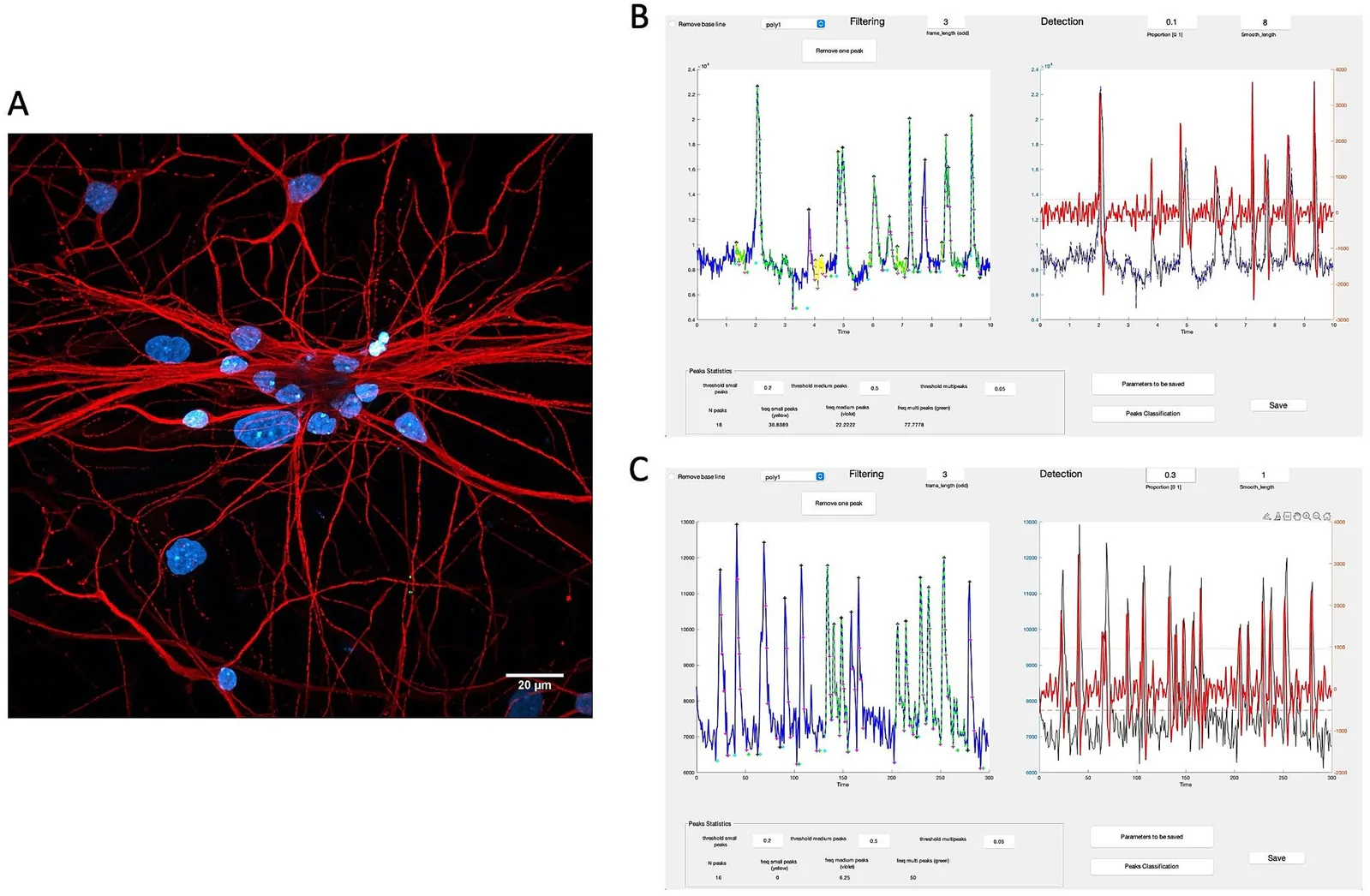
Intracellular Ca^2+^ dynamics in hiPSC-NRs detected by CalDyn. (A) Immunofluorescence staining of healthy neurons, highlighting ß-3 tubulin (red) and DAPI (blue). The scale bar is 20 μm. (B and C) Typical plot illustrating 2 independent and heterogenous intracellular Ca^2+^ handling examples obtained with CalDyn.

To further demonstrate CalDyn in a disease-relevant context, the algorithm was applied to patient-derived hiPSC models carrying pathogenic RyR2 variants associated with catecholaminergic polymorphic ventricular tachycardia and neuro-cardiac manifestations. As detailed in the companion study [23], CalDyn quantified candidate low-amplitude events, alternans, and heterogeneous transient kinetics in RyR2-R169P hiPSC-CMs and hiPSC-NRs, as well as changes observed after treatment with the RyR2-stabilizing compound S107. The biological interpretation of these waveform abnormalities was based on the broader experimental evidence reported in that study. CalDyn classifications alone do not establish that each detected event represents a cytosolic Ca^2+^ leak or an individual Ca^2+^ spark. This application illustrates the use of CalDyn for quantifying subtle and heterogeneous Ca^2+^-transient patterns in disease models.

### Paired comparison of CalDyn and Clampfit for action-potential analysis in hiPSC-CMs

We next evaluated the ability of CalDyn to analyze APs recorded from ventricular-like hiPSC-CMs using the whole-cell current-clamp configuration. The same recordings were analyzed using CalDyn and Clampfit, thereby enabling direct paired comparisons between the two programs. CalDyn automatically detected successive APs and identified the waveform landmarks required to calculate AP duration (APD), amplitude, and maximum upstroke velocity (Fig. 7A). Paired recording-level comparisons were performed for AP duration at 90%, 50%, and 30% of repolarization (APD_90_, APD_50_, and APD₃₀, respectively), AP amplitude, and maximum upstroke velocity (dV/dt_max_; Fig. 7B–F). The paired plots showed that the direction and magnitude of the differences between CalDyn and Clampfit varied among recordings, without a uniform shift across all measurements. The numerical paired-test results and corresponding effect estimates are reported in Table 6. Agreement between CalDyn and Clampfit was further evaluated using Bland–Altman analysis (Supplementary Fig. 5 and Table 6). The mean CalDyn-minus-Clampfit differences were 8.70 ms for APD_90_, −4.80 ms for APD_50_, and −6.60 ms for APD₃₀. Thus, CalDyn produced slightly higher APD_90_ estimates and slightly lower APD_50_ and APD₃₀ estimates on average. The corresponding 95% limits of agreement ranged from −48.85 to 66.25 ms for APD_90_, from −62.94 to 53.34 ms for APD_50_, and from −53.56 to 40.36 ms for APD₃₀ (Table 6). For AP amplitude, CalDyn showed a positive mean bias of 3.80 mV, with 95% limits of agreement ranging from −9.96 to 17.56 mV. Maximum upstroke velocity showed a positive mean bias of 4.40 mV/ms, with 95% limits of agreement ranging from −14.20 to 23.00 mV/ms. These results indicate limited average systematic bias between the two programs, although the width of the limits of agreement shows that larger differences may occur for individual recordings.

**Figure 7:**
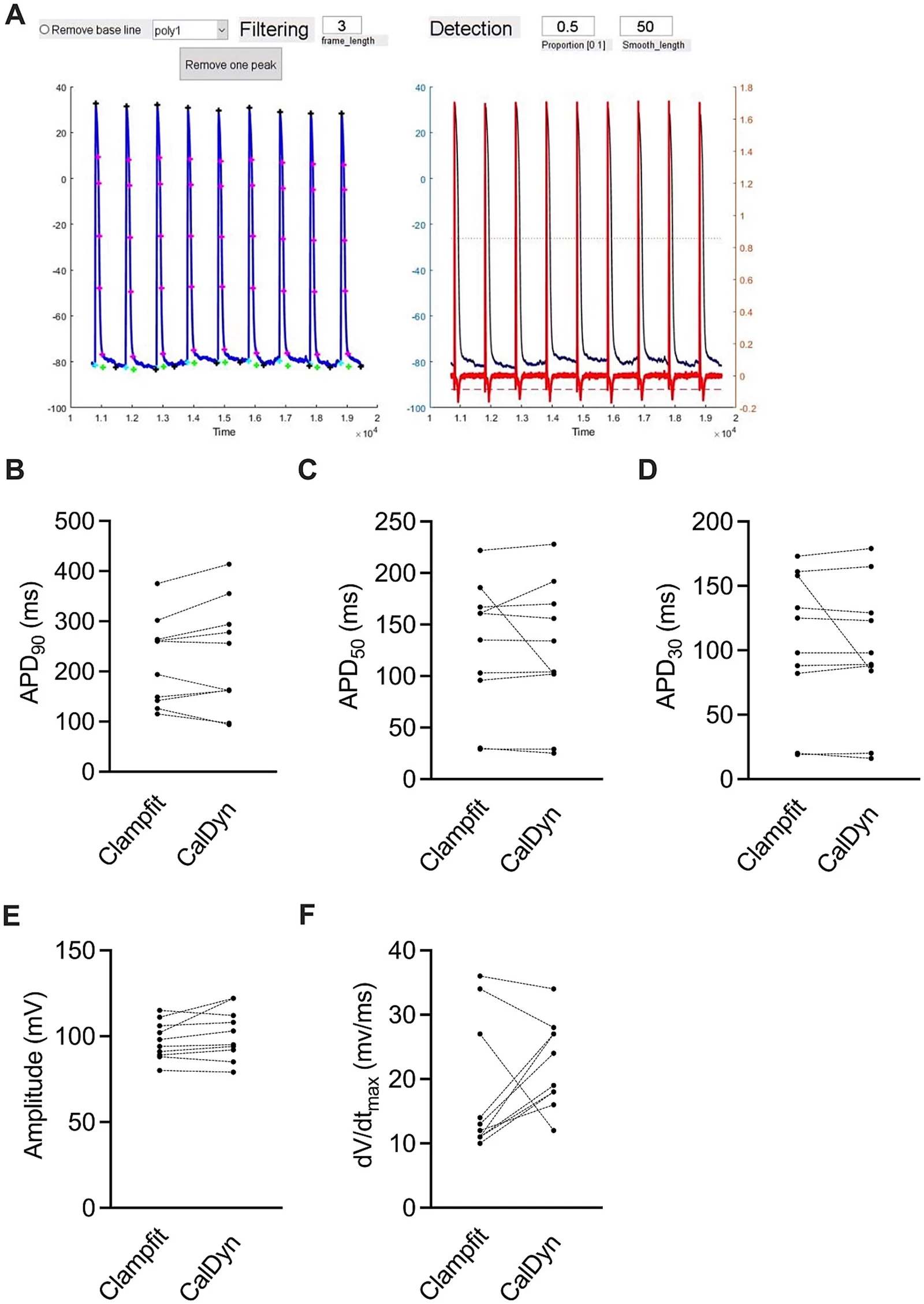
Paired comparison of AP parameters obtained using Clampfit and CalDyn in hiPSC-CMs. (A) Representative CalDyn analysis of successive AP recorded from a ventricular-like hiPSC-CMs in the whole-cell current-clamp configuration. The left panel shows the filtered AP trace together with the waveform landmarks identified by CalDyn for parameter extraction. Black markers indicate detected AP maxima, whereas magenta, cyan, and green markers indicate the positions used to calculate AP duration and waveform boundaries. The right panel shows the AP trace and its filtered derivative used for automated event detection. (B–D) Paired comparisons of AP duration at 90%, 50%, and 30% repolarization, respectively (APD_90_, APD_50_, and APD₃₀). (E) Paired comparison of AP amplitude. (F) Paired comparison of maximum AP upstroke velocity (dV/dt_max_). Each symbol represents one recording, and dotted lines connect measurements obtained from the same recording using the two programs. Bland–Altman analyses of method agreement are presented in Supplementary Fig. 5 and Table 6. APD, action-potential duration; dV/dt_max_, maximum action-potential upstroke velocity.

**Table 6:** Paired comparison and Bland–Altman agreement between CalDyn and Clampfit for AP parameters in healthy control hiPSC-CMs. The same 10 action-potential recordings were analyzed using both CalDyn and Clampfit. Paired comparisons were performed using two-tailed paired *t-*tests. The mean paired difference was calculated as CalDyn - Clampfit; negative values therefore indicate lower estimates obtained using CalDyn, whereas positive values indicate higher estimates obtained using CalDyn. The 95% confidence interval describes the uncertainty surrounding the estimated mean paired difference. The standard deviation of the paired differences describes the dispersion of the individual differences between the two methods. The lower and upper 95% limits of agreement were calculated as the mean paired difference ± 1.96 standard deviations of the paired differences. LoA, limits of agreement; APD_90_, APD_50_, and APD₃₀, AP durations at 90%, 50%, and 30% repolarization, respectively; dV/dt_max_, maximum AP upstroke velocity.

**Parameter**	**Unit**	**Matched pairs, n**	**Mean bias: CalDyn - Clampfit**	**95% CI of mean difference**	**SD of paired differences**	**Lower 95% LoA**	**Upper 95% LoA**	**Paired test**	**Test statistic**	***P*-value**
**APD_90_**	**ms**	**10**	**8.70**	**−12.31 to 29.71**	**29.36**	**−48.85**	**66.25**	**Two-tailed paired *t-*test**	**t(9) = 0.9369**	**.3733**
**APD_50_**	**ms**	**10**	**−4.80**	**−26.02 to 16.42**	**29.66**	**−62.94**	**53.34**	**Two-tailed paired t-test**	**t(9) = −0.5117** ^**1**^	**.6212**
**APD_30_**	**ms**	**10**	**−6.60**	**−23.74 to 10.54**	**23.96**	**−53.56**	**40.36**	**Two-tailed paired t-test**	**t(9) = −0.8711** ^**1**^	**.4063**
**AP amplitude**	**mV**	**10**	**3.80**	**−1.22 to 8.82**	**7.02**	**−9.96**	**17.56**	**Two-tailed paired t-test**	**t(9) = 1.712**	**.1211**
**Maximum upstroke velocity, dV/dt_max_**	**mV/ms**	**10**	**4.40**	**−2.39 to 11.19**	**9.49**	**−14.20**	**23.00**	**Two-tailed paired t-test**	**t(9) = 1.466**	**.1766**

Paired comparisons did not identify statistically detectable systematic differences between CalDyn and Clampfit for APD_90_ (*t*(9) = 0.9369, *P* = .3733), APD_50_ (*t*(9) = −0.5117, *P* = .6212), APD₃₀ (*t*(9) = −0.8711, *P* = .4063), action-potential amplitude (*t*(9) = 1.712, *P* = .1211), or maximum upstroke velocity (*t*(9) = 1.466, *P* = .1766; Fig. 7B–F and Table 6). The confidence intervals for all mean paired differences included zero. Because a non-significant paired test does not establish equivalence, agreement between the methods was additionally evaluated using Bland–Altman analysis. Visual inspection of the Bland–Altman plots identified isolated recording-level discrepancies for APD_50_, APD₃₀, action-potential amplitude, and maximum upstroke velocity (Supplementary Fig. 5). These differences may reflect software-specific definitions of waveform landmarks, AP boundaries, or derivative-based measurements. Collectively, the analyses show that CalDyn provides group-level AP parameter estimates broadly comparable with those obtained using Clampfit, while also defining the extent of recording-level disagreement between the two methods. The observed limits of agreement should therefore be considered when interpreting measurements from individual recordings, and the programs should not be assumed to be interchangeable without appropriate quality control.

### CalDyn is compatible to monitor cardiac APs recording by optical mapping

We tested CalDyn’s ability to analyze optical voltage from *ex vivo* Langendorff-perfused guinea pig hearts using the voltage-sensitive probe RH237. We compared the properties obtained by CalDyn and BV Workbench provided with the equipment (Fig. 8A). We focused on temporal parameters as intensity in BV Workbench is normalized in arbitrary units of fluorescence. We found that peaks in the signal are easily detected and measured in CalDyn (Fig. 8B). Average measurements over 24 points were compared between CalDyn and BV Workbench. Values are in agreement except the diastolic interval (Table 7). But this last parameter is dependent on its definition, which is different between CalDyn and BV Workbench.

**Figure 8:**
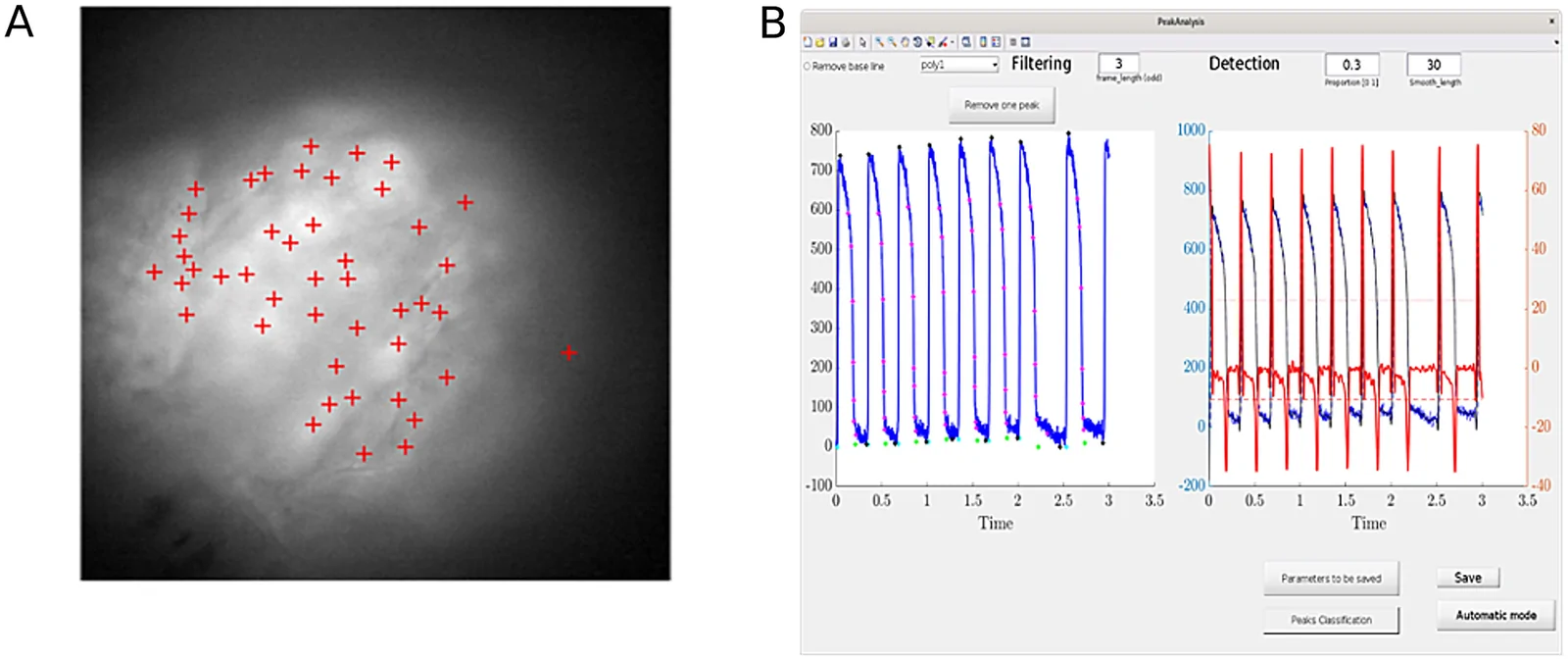
Optical mapping recording using RH237 fluorescent probe.

**Table 7:** Comparison of parameters between BV Workbench et Caldyn.

	**Peak Interval (ms)**	**Rise time (ms)**	**80% Decay time (ms)**	**Diastolic interval (ms)**
BV Workbench	334.0 ± 3.5	20.0 ± 2.9	159.1 ± 2.4	163.0 ± 1.0
CalDyn	336.0 ± 3.8	18.0 ± 4.0	156.0 ± 4.0	140.0 ± 2.0

The theoretical definition of the diastolic interval is the duration of the rest phase, i.e. the time between the end of a peak and the start of the next one.

In Workbench, DI is defined in the following way: taking 2 consecutive APs, the software marks the activation and 80% restitution time points of each AP (actT1 and restT1 for AP1; actT2 and restT2 for AP2). DI = (actT2 - restT1). It is affected by the chosen APD restitution threshold (50%, 80%, 90%) and by the definition of the activation point (foot, half max, max dF/dt).

CalDyn instead gives a value much closer to the theoretical definition (and not dependent on the choice of the APD and activation point), as it is the difference between the value of the points marked in green and cyan for two consecutive peaks, which really are the base of the peaks (see their definition in the description of the CalDyn algorithm section).

Because BV Workbench’s operational DI is intrinsically tied to a chosen APD-restitution threshold and activation-point definition, its absolute value shifts if a different convention is chosen, even for identical underlying data. CalDyn’s DI, computed directly from the fitted boundary points, is architecture-independent and tracks the theoretical definition more closely, which we consider the more standard-conforming of the two, but this also means CalDyn’s DI values are not numerically interchangeable with prior BV Workbench-based literature values using the 80%-restitution convention. We recommend that cross-study DI comparisons always state which definition/threshold was used, since this difference could otherwise be mistaken for a genuine physiological effect.

### CalDyn’s detection accuracy validated against expert reference

The comparisons above establish agreement with existing software, but agreement between two automated or semi-automated methods does not by itself establish which output is correct. To address this directly, we established an expert-reference benchmark: an expert manually annotated peak onset, maximum, and offset (one click each, snapped to the nearest data sample) across 142 peaks spanning all six acquisition modalities reported in this manuscript (healthy hiPSC-CMs, arrhythmic PMVT hiPSC-CMs, paced skeletal myotubes, patch-clamp APs, hiPSC-NRs, and optical mapping), using a purpose-built annotation tool. CalDyn’s automated detection, run with the exact parameters used for each modality throughout this manuscript, was then scored against these annotations by matching each detected peak to the nearest expert reference event within an adaptive per-trace tolerance (25% of that trace’s local inter-peak interval), and computing sensitivity, precision, F1 score, and timing/amplitude error (Table 8).

**Table 8:** CalDyn detection performance against expert-reference annotations. Median absolute timing error, expressed as a percentage of that trace’s local inter-peak interval so it is comparable across modalities with very different native timescales. Offset-timing error is not tabulated here (median 27.9% overall) (see Discussion).

Modality	n peaks	Sensitivity	Precision	F1	Onset error^*^	Peak error^*^	Amplitude error
Healthy hiPSC-CMs	15	100%	100%	1.00	1.3%	1.1%	0.4%
Arrhythmic hiPSC-CMs	35	80%	93%	0.86	4.6%	0.9%	1.3%
Paced myotubes	8	100%	100%	1.00	1.2%	~0%	0%
AP (patch-clamp)	16	100%	100%	1.00	0.9%	0.4%	3.2%
hiPSC-NRs (2 recordings)	52	100%	68%–96%	0.76–0.98	4.4%–6.1%	~0%	0%
Optical mapping	16	94%	100%	0.97	1.2%	2.3%	0.3%
All modalities combined	142	94.4%	89.9%	0.92	1.3%	~0%	~0%

Peak position and amplitude were recovered essentially exactly (median error ≈0%), consistent with CalDyn defining a peak as the true local maximum of the filtered signal, the same feature a human annotator naturally identifies. Onset timing was accurate (1%–6% of the local inter-peak interval). Offset timing was markedly less precise (median 27.9% overall, range 11%–46%); we interpret this as reflecting genuine ambiguity in defining “where a decay ends” on an asymptotically-decaying curve, for a human annotator as much as for the algorithm, rather than a specific shortcoming of CalDyn’s boundary-fitting method. Detection recall (sensitivity) was ≥94% in every modality except arrhythmic hiPSC-CMs (80%, i.e. CalDyn missed a fraction of the events an expert annotator identified), and precision was lowest for hiPSC-NRs (68%–96%: CalDyn flagged more small-amplitude events than the annotator marked). We discuss both findings as genuine modality-specific limitations rather than presenting only the favorable metrics.

#### Controlled synthetic validation of filter-induced bias

The expert-reference benchmark above uses real recordings, which cannot isolate individual sources of measurement error from one another. To test directly whether CalDyn’s sequential Savitzky–Golay/LOESS filtering biases peak position, amplitude, or kinetics, we generated synthetic signals with an analytically known true peak time and amplitude (an asymmetric rise/decay shape, 30 ms rise time constant, 200 ms decay time constant) and independently varied noise level, sampling rate, baseline drift, and inter-event spacing, holding the other three factors fixed at a typical value for each sweep (20 repetitions per condition; Table 9).

**Table 9:** CalDyn detection performance on synthetic signals with known ground truth, with one factor varied at a time. Timing error is expressed as a percentage of the decay time constant so it is comparable across conditions.

Factor varied	Range tested	Detection rate	Timing error (% of decay time)	Amplitude error (%)
Noise (SNR)	1%–40% of peak amplitude	95%–100%	0.6%–5.6%, no clear trend	0.3% → 8.4%, increases with noise
Sampling rate	20–500 Hz	85%–100%	19.4% at 20 Hz; ≤3% at ≥50 Hz	0.5%–2.0%, no clear trend
Baseline drift (uncorrected)	0%–50% of peak amplitude	80%–100%	0.6%–4.4%, no clear trend	1.2% → 19.5%, increases with drift
Inter-event spacing	1.5–10× decay time	100%	0.6%–5.6%, no clear trend	0.5%–1.3%, no clear trend

At every spacing tested (1.5× to 10× the decay time constant), both peaks were correctly resolved as two distinct events, rather than merged into one or one being missed, in 95%–100% of trials. Every metric degraded gradually and monotonically as conditions worsened (more noise, coarser sampling relative to the signal’s own kinetics, larger uncorrected drift), which is the expected behavior of a peak-detection method applied to progressively harder data, and we found no discontinuity or bias signature specific to the LOESS filtering step. The one condition with a substantially larger timing error, 20 Hz sampling, corresponds to a rise time spanning barely more than half a sample interval at that rate: an undersampling limitation relative to the signal’s own kinetics rather than a filtering artifact. The amplitude error at high uncorrected drift (up to 19.5%) is, similarly, not an algorithmic limitation but a direct quantitative illustration of why CalDyn provides an optional baseline-correction step in the first place; this sweep did not use it.

#### Detection-parameter sensitivity analysis

The comparisons above use each dataset’s own default detection parameters throughout. To test how sensitive CalDyn’s detection outcomes are to those choices, we took the same 142-peak expert reference used for Table 8 and re-scored detection after independently scaling each of the three detection parameters (filter length param_filter, smoothing span Smoothness, derivative-threshold proportion prop) to 0.5×, 0.75×, 1× (default), 1.5×, and 2× its default value, one parameter at a time, holding the other two at their defaults, across all seven expert reference datasets (Table 10). This is distinct from the classification thresholds (th_smpks/th_medpks/th_multi) discussed above, whose effect is an exact, deterministic arithmetic comparison rather than an empirical detection outcome.

**Table 10:** Detection-parameter sensitivity, scored against the same 142-event expert reference as Table 8, with each detection parameter varied individually from 0.5- to 2-fold of its default value across the seven expert-reference datasets. Values are expert-reference-event-weighted averages across datasets. The 1-fold column reproduces the overall results in Table 8; the small numerical difference in precision reflects averaging per-dataset precision rather than pooling raw event counts and does not represent a difference in detections.

Parameter	0.5×	0.75×	1× (default)	1.5×	2×
Filter length - Sensitivity / Precision / F1	94.4% / 91.7% / 0.92	94.4% / 91.7% / 0.92	94.4% / 91.7% / 0.92	94.4% / 92.5% / 0.93	93.0% / 91.7% / 0.92
Smoothing span - Sensitivity / Precision / F1	97.2% / 81.0% / 0.87	95.8% / 87.2% / 0.90	94.4% / 91.7% / 0.92	90.1% / 92.7% / 0.91	85.9% / 97.5% / 0.91
Threshold proportion - Sensitivity / Precision / F1	96.5% / 75.5% / 0.83	94.4% / 84.2% / 0.88	94.4% / 91.7% / 0.92	88.7% / 99.4% / 0.93	75.4% / 100% / 0.82

All three parameters degrade gracefully and in the expected direction rather than erratically: loosening a parameter (shorter filter, smaller smoothing span, lower threshold proportion) trades precision for sensitivity, and tightening it does the reverse, with F1 remaining within 0.82–0.93 across the entire 0.5 × −2× range tested for every parameter. Filter length was the most robust of the three (F1 0.92–0.93 throughout). The threshold proportion was the most sensitive parameter at its extremes: doubling it raised precision to 100% but lowered sensitivity to 75.4% pooled (one dataset, healthy hiPSC-CMs, dropped from 100% to 13% sensitivity at 2× its default prop), which is the expected consequence of a much stricter derivative threshold excluding genuine but lower-amplitude peaks, not an anomaly. We take this as evidence that CalDyn’s default parameters, each tuned per modality against visual inspection during development, sit in a stable region of parameter space rather than a narrow, fragile optimum, while also showing that, as with any threshold-based detector, pushing a parameter far enough away from its appropriate range will degrade results, which is why per-modality defaults and ranges are documented.

## Discussion

In this work, we have designed CalDyn, a high-throughput automated algorithm for monitoring of intracellular Ca^2+^ dynamics in any cells that exhibit intracellular Ca^2+^ dynamics. The signal-processing approaches underlying CalDyn, including Savitzky–Golay filtering, LOESS smoothing, and derivative-based peak detection, are well-established and are not presented here as novel methodological concepts. Rather, the contribution of CalDyn lies in their integration into a unified analysis framework designed to address several practical limitations of existing approaches. Specifically, CalDyn combines dual filtering with derivative-based thresholding to enable robust analysis across heterogeneous and non-stationary signals without the need for parameter re-tuning for individual recordings, including fluctuating-baseline hiPSC-NR traces and irregular arrhythmic transients. In addition, CalDyn incorporates a rule-based classification of small, medium, and multipeak events, a feature not implemented in the comparator tools discussed below. The framework further provides a single analysis workflow applicable across three acquisition modalities, Ca^2+^ imaging, patch-clamp current-clamp recordings, and optical mapping, while generating a consistent spreadsheet-based output. Finally, its GUI and standalone executable facilitate use without programming expertise or a MATLAB license, thereby making the analysis workflow accessible to a broader range of users. The analysis of Ca^2+^ signals in various cellular contexts has been a topic of intense research due to the crucial role of Ca^2+^ ions in regulating a multitude of intracellular processes [24]. However, the preliminary interpretation of Ca^2+^ signaling data is commonly qualitative and to some extent subjective. Nonetheless, by applying mathematically well-defined criteria and maintaining consistent parameters, it becomes feasible to conduct repeatable and standardized analyses. This consistent quantitative analysis significantly diminishes subjective aspect, ensuring more reliable and objective interpretations of the data. In the context of Ca^2+^ signal analysis, the development of tools like CalDyn, designed to decipher diverse and complex Ca^2+^ signaling patterns, offers researchers valuable insights into a range of cellular phenomena. Furthermore, our work suggests the versatility of CalDyn to analyze voltage and Ca^2+^ parameters in an array of cellular and tissue models. This encompasses APs obtained through the patch-clamp recordings. This adaptability provides a comprehensive view of cellular behavior, allowing researchers to correlate data from different sources. The use of software automation eliminates manual user input, reducing the potential for user error and bias. This standardized approach ensures the provision of Ca^2+^ transient data that is consistent and comparable across different cells and experimental conditions.

Agreement between analytical tools should be distinguished from analytical accuracy, as differences in preprocessing, event definitions, waveform-boundary criteria, and calculation methods can produce systematic differences even when the same recordings are analyzed. Paired comparisons with PeakInspector demonstrated that CalDyn can identify and characterize Ca^2+^ transients in both healthy and arrhythmic hiPSC-CMs, while revealing differences in several derived parameters. In healthy hiPSC-CMs, CalDyn yielded modestly higher normalized amplitudes, lower principal-transient frequencies, and substantially higher maximum rise-rate estimates than PeakInspector. In PMVT recordings, differences were also observed in normalized amplitude, principal-transient frequency, candidate multicomponent Ca^2+^ transient frequency, and decay time. Bland–Altman analyses further showed that relatively small mean biases for some parameters could coexist with appreciable differences at the level of individual cells or recordings. These findings indicate that, although both tools characterize Ca^2+^ dynamics, their outputs should not be considered directly interchangeable because the resulting measurements depend on the analytical definitions and algorithms implemented by each program.

The pronounced difference in dF/dt_max_ estimates illustrates the importance of these operational definitions. PeakInspector estimates rise rate from transient amplitude and rise time, whereas CalDyn calculates the slope directly from the ascending phase of the transient. Although both parameters characterize the rising phase of the Ca^2+^ transient, they are mathematically distinct. CalDyn therefore provides a direct waveform-derived estimate of the maximum fluorescence rise rate rather than an estimate derived from amplitude and rise time. Nevertheless, dF/dt_max_ should not be interpreted as a direct measurement of RyR2-mediated Ca^2+^ release, as fluorescence kinetics are also influenced by cytosolic buffering, probe kinetics, sampling rate, normalization, and spatial averaging.

The AP comparison showed smaller average systematic differences between CalDyn and Clampfit. Mean biases for APD_90_, APD_50_, APD₃₀, action-potential amplitude, and maximum upstroke velocity were limited relative to the observed measurement ranges. Paired tests did not identify statistically detectable mean differences between the programs. Nevertheless, the Bland–Altman limits of agreement showed that larger differences could occur for individual recordings. Thus, a non-significant paired comparison should not be interpreted as demonstrating equivalence. Visual inspection and consistent waveform definitions remain necessary when comparing CalDyn-derived measurements with previously published Clampfit results [13].

The comparison with BV Workbench further demonstrated the applicability of CalDyn to optical-mapping data. Peak interval, rise time, and decay-time estimates were broadly comparable, whereas diastolic interval differed because the two programs use distinct waveform landmarks. BV Workbench calculates diastolic interval from selected activation and restitution thresholds, whereas CalDyn calculates the interval between fitted waveform boundaries. Neither measurement should be compared across studies without explicitly reporting the definition, activation point, and restitution threshold used. This distinction is particularly relevant in optical-mapping studies, in which activation and repolarization metrics depend on signal processing and threshold conventions [14, 15].

The ability of CalDyn to investigate Ca^2+^ signals in immature cells derived from stem cells, garners substantial interest in the field. These cells often exhibit unique Ca^2+^ dynamics associated with their developmental processes. We have shown that CalDyn is particularly well-suited for this task due to its precision and adaptability in handling complex, evolving Ca^2+^ patterns. CalDyn, in particular, facilitates powerful data analysis and measurement of various parameters, such as the standard deviation of the decay slope. This capability helps in detecting different dynamics of Ca^2+^ handling during peak decay.

To the best of our knowledge, there is no similar easy-to-process and automatic tool providing simple spreadsheet format output. Our data indicate that CalDyn has exclusive features in analyzing irregular Ca^2+^ signals. In particular, due to their immature and diverse phenotype, hiPSC-NRs exhibit heterogeneous Ca^2+^ release associated with several intracellular Ca^2+^ release channels, mainly IP_3_R and RyR [25]. These irregular Ca^2+^ signals can be particularly challenging to analyze, but they hold essential information about cellular activities. CalDyn’s strength lies in its capability to dissect and interpret these irregular signals effectively, making it instrumental in the study of cell types exhibiting atypical Ca^2+^ responses.

CalDyn can detect and classify candidate low-amplitude diastolic and multicomponent Ca^2+^ events, making it useful for examining heterogeneous waveform patterns in pathological cardiomyocyte recordings. Such events may be biologically relevant because prior studies have shown that sarcoplasmic-reticulum Ca^2+^ leak can occur through discrete spark-like release events and can contribute to triggered activity and arrhythmogenesis [1, 26]. However, the events identified in the present PMVT recordings were classified from whole-cell fluorescence waveforms and were not independently confirmed by spark-resolved imaging or another orthogonal method. They should therefore be described as candidate low-amplitude diastolic or multicomponent events rather than as confirmed Ca^2+^ sparks or direct measurements of sarcoplasmic-reticulum Ca^2+^ leak. Previous findings in CPVT and dystrophin-deficient hiPSC-CMs support the biological plausibility of altered diastolic Ca^2+^ handling [11], but do not establish the identity of each CalDyn-detected event.

Moreover, previous work has shown that FKBP12.6 binding to RyR2 modulates Ca^2+^ spark amplitude [27]. In the previously characterized PMVT RyR2-H29D model, FKBP12.6 depletion was associated with increased diastolic Ca^2+^ leak, and pharmacological stabilization of FKBP12.6 binding reduced this phenotype [9]. Complementary single-channel recordings also showed increased open probability of recombinant RyR2-H29D channels under diastolic conditions [28]. These independent observations support the biological plausibility of altered diastolic Ca^2+^ handling in this model, but they do not establish that every candidate low-amplitude or multicomponent event detected by CalDyn in the present recordings represents an individual Ca^2+^ spark. Candidate multicomponent Ca^2+^ transients in PMVT hiPSC-CMs were identified more frequently by CalDyn than by PeakInspector. This difference demonstrates distinct detection and classification behavior between the programs, rather than proving that one method is more biologically accurate. The additional CalDyn-detected events are consistent with heterogeneous intracellular Ca^2+^ cycling and may be compatible with spontaneous SR Ca^2+^ release associated with dysfunctional RyR2 regulation. That interpretation is biologically plausible in light of previous reports of discrete diastolic release events [26]. FKBP12.6-dependent Ca^2+^ leak in the RyR2-H29D model [9], increased RyR2 open probability in single-channel recordings [28], and pharmacological responses reported in a companion RyR2 study [23]. Nevertheless, the present waveform analysis does not independently establish that each candidate low-amplitude or multicomponent event represents a Ca^2+^ spark or a direct measure of sarcoplasmic-reticulum Ca^2+^ leak.

The companion RyR2-R169P study [23], provides an additional disease-model application of CalDyn. In that study, CalDyn quantified candidate low-amplitude events, alternating transients, and heterogeneous release patterns in hiPSC-CMs and hiPSC-NRs, together with changes after treatment with S107. These waveform measurements should be interpreted alongside the orthogonal experimental evidence reported in the companion study rather than as independent proof that each CalDyn-detected event is a cytosolic Ca^2+^ leak. The LMNA p.H222P and CTX-1B cardiac-sheet datasets provide additional descriptive applications of CalDyn in multicellular preparations. In the LMNA dataset, CalDyn extracted ROI-level measurements of transient periodicity, multipeak-event frequency, rise time, and decay time from LMNA p.H222P and gene-corrected isogenic-control cardiac sheets. In the pharmacological dataset, CalDyn extracted transient amplitude, inter-transient period, rise time, and decay time from vehicle- and CTX-1B-exposed cardiac sheets. Because the ROIs were nested within only four independent sheets in each experiment, these ROI-level distributions are presented as application examples rather than as confirmatory genotype or treatment comparisons. The datasets demonstrate the feasibility of applying CalDyn to multicellular genetic and pharmacological models but do not establish population-level differences.

To our knowledge, this is the first automated solution that discerns these events without requiring specialized expertise in ECC analysis. To compare, SparkMaster 2 [29] is an open-source tool that offers an enhanced spark-detection algorithm that significantly improves accuracy in spark detection, outperforming the previous SparkMaster [30]. SparkMaster 2 provides a wider array of features and, despite increased complexity, offers faster data analysis. However, its application is confined to detecting line-scan Ca spark imaging.

In the context of high-throughput automated algorithms for monitoring intracellular Ca^2+^ dynamics, it is essential to consider the strengths and limitations of analytical tools such as SIMA [3] and CaImAn [5] in relation to the described work. Both SIMA and CaImAn are script-based tools utilized for processing and analyzing Ca^2+^ imaging datasets. Both address challenges related to Ca^2+^ imaging data analysis, including motion correction and neural activity identification. However, it is imperative to note potential limitations. SIMA may present challenges in terms of complex installation and setup, as well as limited user-friendly documentation, which may hinder its accessibility to researchers with varying levels of programming experience. Conversely, while CaImAn offers automated and scalable methods for Ca^2+^ imaging data analysis, users may find it challenging to optimize parameters for specific datasets, potentially creating a steep learning curve for new users. While primarily designed for two-photon Ca^2+^ imaging, EZCa^2+^ has the potential to be compatible with other types of dynamic fluorescence data, such as voltage indicators and neurotransmitter release probes, making it a versatile tool for a broader range of imaging experiments [31].

In contrast to the tools mentioned, PeakInspector primarily focuses on the detection and analysis of Ca^2+^ transients or spikes. Our data showed that PeakInspector provides values for each peak per cell, resulting in significantly more data, while CalDyn provides the average value of each parameter per cell. PeakInspector requires manual identification of candidate low-amplitude diastolic events and candidate multicomponent Ca^2+^ transients, which can introduce biases, whereas CalDyn does not. In PeakInspector, df/dt is calculated manually using the absolute amplitude divided by the ascending time, while CalDyn uses the mean ascending slope. Our data indicated that CalDyn offers a more comprehensive analysis, including the study of irregular signals, action potentials, and extracellular field potentials.

Table 11 summarizes these comparisons. We have not run head-to-head benchmarks against SparkMaster 2, SIMA, CaImAn, or EZCa^2+^ on the same data, so we make no claim of quantitative superiority over them, the comparison below is qualitative (modality coverage, classification features, accessibility) rather than a performance ranking.

**Table 11:** Qualitative comparison of CalDyn with related analysis tools.

Tool	Modality	Peak/event classification	GUI	License
CalDyn	Ca^2+^ imaging, patch-clamp AP, optical mapping	small/medium/multipeak, rule-based	Yes (MATLAB app or standalone .exe)	Free
PeakInspector	Ca^2+^ imaging (line-scan/whole-cell)	Manual	Yes (Python)	Free
SparkMaster 2	Line-scan Ca^2+^ sparks only	Spark detection only	Yes	Free
SIMA / CaImAn	Ca^2+^ imaging	None	Script-based	Free
EZCa^2+^	2-photon Ca^2+^ imaging	None	Yes	Free
Clampfit	Patch-clamp	Event sorting	Yes	Paid (pCLAMP suite)
BV Workbench	Optical mapping	Peak detection	Yes	Paid

We chose MATLAB as the platform for developing CalDyn due to several key advantages it offers. Unlike open-source alternatives like Java or Python, MATLAB is distinguished for its stability and computational efficiency, particularly in handling intricate computations. This characteristic ensures the reliability and consistency of CalDyn as a tool for Ca^2+^ signal analysis over time. MATLAB’s robustness and ability to handle complex calculations effectively reinforce the dependability of CalDyn, providing a stable and consistent environment for Ca^2+^ and AP signal analysis. Specifically, MATLAB’s numerical computation environment supports efficient batch analysis of electrophysiological and Ca^2+^-transient signals. CalDyn runs on base MATLAB alone, with no additional toolboxes required (see Availability statement), while still benefiting from the platform-level advantages described below. Its native support for matrix operations and parallelization ensures numerical stability, particularly for edge cases such as detecting candidate low-amplitude or multicomponent Ca^2+^ events, which can then be interpreted in the context of the experimental model and appropriate biological controls.

MATLAB’s App Designer enables the development of intuitive graphical user interfaces (GUIs) with minimal coding overhead, streamlining accessibility for non-specialists. This contrasts with Python’s GUI frameworks (e.g. Tkinter, PyQt), which require extensive customization and technical expertise. MATLAB’s closed ecosystem also guarantees backward compatibility across versions, ensuring long-term reproducibility, a critical factor for clinical and translational research involving patient-derived cells. While Python offers broader accessibility, its open-source nature introduces risks of breaking changes in dependencies (e.g. NumPy, SciPy), which could destabilize workflows over time. Additionally, MATLAB’s seamless integration with legacy tools (e.g. Clampfit, BV Workbench) and file formats (e.g. ABF, TIFF) simplifies interoperability with gold-standard platforms for patch-clamp and optical mapping data. Finally, MATLAB’s integrated development environment (IDE) accelerates algorithm prototyping and debugging, which was instrumental in refining CalDyn’s peak detection and baseline correction algorithms. Though Python-based solutions may expand accessibility, MATLAB’s specialized capabilities, precision, stability, and user-friendly design make it the pragmatic choice for CalDyn’s mission-critical applications in biomedical signal analysis.

Furthermore, we have generated a standalone Windows version of CalDyn, which eliminates licensing barriers by leveraging MATLAB Runtime. This precompiled executable allows users without a full MATLAB license to run CalDyn seamlessly on Windows systems, ensuring broad accessibility while preserving the computational stability inherent to MATLAB’s closed ecosystem. By decoupling the software from MATLAB licensing requirements, we aim to democratize access for researchers in institutions without MATLAB subscriptions, particularly in clinical and translational settings where licensing constraints may hinder adoption.

### Limitations

Several limitations of the present work should be considered when interpreting our results. First, some of our validation comparisons involve small sample sizes; these are best read as feasibility/agreement demonstrations rather than adequately powered equivalence studies. Second, the peak-classification thresholds (m, Q, M) are recalculated independently for every analyzed trace rather than pooled across a group or fixed from an external reference; a “small” peak is therefore only guaranteed to be small relative to that trace’s own peak-height distribution, which should be kept in mind when comparing classification frequencies between experimental groups. Third, the peak-boundary algorithm assumes a faster rising phase and slower descending phase, which holds for the Ca^2+^ transients, action potentials, and optical-mapping voltage signals validated here, but has not been tested on signal types with symmetric or reversed kinetics. Fourth, our expert-reference benchmark surfaced two modality-specific gaps worth stating plainly: recall was lower in arrhythmic hiPSC-CM recordings (80%, CalDyn missed some expert-identified events) and precision was lower in hiPSC-NR recordings (68%–96%, CalDyn flagged more small-amplitude events than the annotator marked), the latter being a direct consequence of the sensitivity to low-amplitude events that we present elsewhere as a strength, which is worth holding in tension rather than presenting one-sidedly. Fifth, the detection-parameter sensitivity sweep shows graceful, expected-direction degradation (F1 0.82–0.93 across 0.5 × −2× of default for all three detection parameters), but it necessarily only tests parameter values around each dataset’s own already-reasonable default; it does not establish how detection would behave starting from a poorly chosen default, nor does it test the three detection parameters in combination rather than one at a time. The classification thresholds are not part of this open item, since classification is a deterministic labeling rule applied to already-validated peak heights rather than an estimate, and so has no separate accuracy to characterize (see CalDyn Algorithm, Peak classification). Candidate low-amplitude diastolic and multicomponent events are analytical waveform classes. CalDyn-only events in the present recordings were not independently confirmed by spark-resolved imaging or another orthogonal method and should not be interpreted as direct measurements of sarcoplasmic-reticulum Ca^2+^ leak or as confirmed individual Ca^2+^ sparks. Finally, validation against SparkMaster 2, SIMA, CaImAn, and EZCa^2+^ is qualitative; no head-to-head quantitative benchmark against these tools has been performed.

In conclusion, CalDyn provides an accessible and configurable workflow for automated analysis of heterogeneous Ca^2+^ and electrophysiological time-series signals across the evaluated modalities. The platform supports consistent parameter extraction, batch processing, visual quality control, and spreadsheet-compatible export. Candidate low-amplitude and multicomponent events detected by CalDyn should be interpreted as analytical waveform classes requiring appropriate parameter selection and, where biological identity is important, orthogonal experimental confirmation.

**Key Points**

- CalDyn is a MATLAB-based automated platform for analyzing intracellular Ca^2+^ dynamics and electrophysiological time-series signals. The workflow was evaluated using healthy and disease-associated hiPSC-derived cardiomyocytes, hiPSC-derived neurons, primary human skeletal myotubes, cardiac sheets, patch-clamp action potentials, and *ex vivo* optical-mapping recordings.
- CalDyn integrates established signal-processing approaches into a unified and configurable workflow. The platform combines Savitzky–Golay filtering, LOESS-assisted derivative detection, optional baseline correction, waveform-boundary fitting, rule-based event classification, batch processing, visual quality control, and spreadsheet-compatible data export.
- Matched comparisons with PeakInspector and Clampfit identified both agreement and parameter-specific differences between analytical methods. Bland–Altman analyses quantify systematic bias and recording-level limits of agreement, emphasizing that outputs from different programs should not automatically be considered interchangeable.
- CalDyn supports the detection and characterization of heterogeneous waveform patterns, including principal transients, low-amplitude candidate events, and multicomponent transients. These classifications are trace-relative analytical labels and require appropriate parameter selection and biological interpretation.
- Detection performance was evaluated independently against 142 expert-annotated events across six acquisition modalities. CalDyn achieved 94.4% sensitivity, 89.9% precision, and an overall F1 score of 0.92. Peak-maximum localization and amplitude estimation were accurate for matched events, whereas offset estimation and performance in arrhythmic cardiomyocyte and neuronal recordings were less precise.
- Synthetic-signal and parameter-sensitivity analyses defined important operating conditions and limitations. Detection performance was broadly stable across moderate variation of the principal detection parameters, while low sampling rates, substantial noise, and uncorrected baseline drift increased measurement error.
- CalDyn is designed to support accessible and reproducible analysis. The software provides documented parameters, automated graphical outputs, spreadsheet-compatible results, versioned source code and example datasets on GitHub, millisecond-scale computation under the reported benchmark conditions, and a standalone Windows executable for users without a MATLAB license.

## Supplementary Material

Supplementary_Materials_bbag602

## Data Availability

Caldyn is freely accessible with a quick starting guide on GitHub: ‘https://github.com/heleneayari/CalDyn’, under the GPL-3.0 license, release V1.2 (commit e476160). CalDyn was developed and tested in MATLAB R2022a and requires only base MATLAB, no additional toolboxes (confirmed with MATLAB’s own dependency analyzer, matlab.codetools.requiredFilesAndProducts). The standalone Windows executable requires MATLAB Runtime matching the compiled MATLAB version (see the GitHub Install guide); example data for every modality reported in this manuscript are provided under Data/ in the repository. Processing time was benchmarked on synthetic Ca^2+^-transient-like traces (MATLAB R2022a, 16-core workstation, 33.5 GB RAM): 1.3–8.4 ms per trace once batch size ≥50, scaling with both trace length (1000–20 000 points tested) and dataset size, comfortably supporting the sub-second-per-series claim above even before batching gains. Full benchmark code and results are provided under Benchmark in the repository. Beyond runtime, we quantified export cost, memory scaling, and batch failure rate on the same benchmarking machine. For a representative 5000-point trace, compute-only processing (filtering and parameter extraction) took 17 ms, while creating the results figure and exporting it to PNG took an additional 229 ms; export/rendering therefore dominates single-trace latency even though total time remains sub-second. Process memory was measured directly as the MATLAB process memory delta for batches of 1–250 traces (1000-point traces) and fitted with a linear model (≈4.0 MB per added trace); extrapolating this fit, a single-file batch of 500, 1000, 2000 and 5000 traces is projected to use ~1.3, 3.3, 7.3, and 18.9 GB respectively, comfortably within the 33.5 GB benchmarking machine for batches up to a few thousand traces but approaching its RAM ceiling for the largest batch sizes tested; machines with more available memory, or batching very large datasets across multiple files, would extend this ceiling further. Batch robustness was assessed on five categories of pathological single-trace inputs (flat signal, pure noise, single spike, abnormally short trace, and trace with dropout/NaN values; 50 trials per category, 250 trials total): 200/250 traces (80%) were processed without a thrown error; all 50 failures were confined to the abnormally short-trace category, where the fixed-length smoothing filter cannot be applied to a signal shorter than its window and a specific, identifiable error is raised rather than a silent or incorrect result. Full benchmark code and results, including the export-timing, memory-scaling, and failure-rate data above, are provided under Benchmark in the repository.
