# Supplementary material for: CalDyn: an advanced high-throughput automated algorithm for monitoring intracellular calcium dynamics and electrophysiological signals in patient-derived cells
Source: Brief Bioinform. 2026 Nov 7;27(6):bbag602. doi: 10.1093/bib/bbag602 (PMC13744280; doi:10.1093/bib/bbag602)
Supplement: Supplementary file 1 [file supplementary_materials_bbag602.zip › Supplementary_Materials_bbag602/CalDyn_supplementary_material_R2_v3_bbag602.pdf]

Supplemental Figures
Supplemental Figure 1:

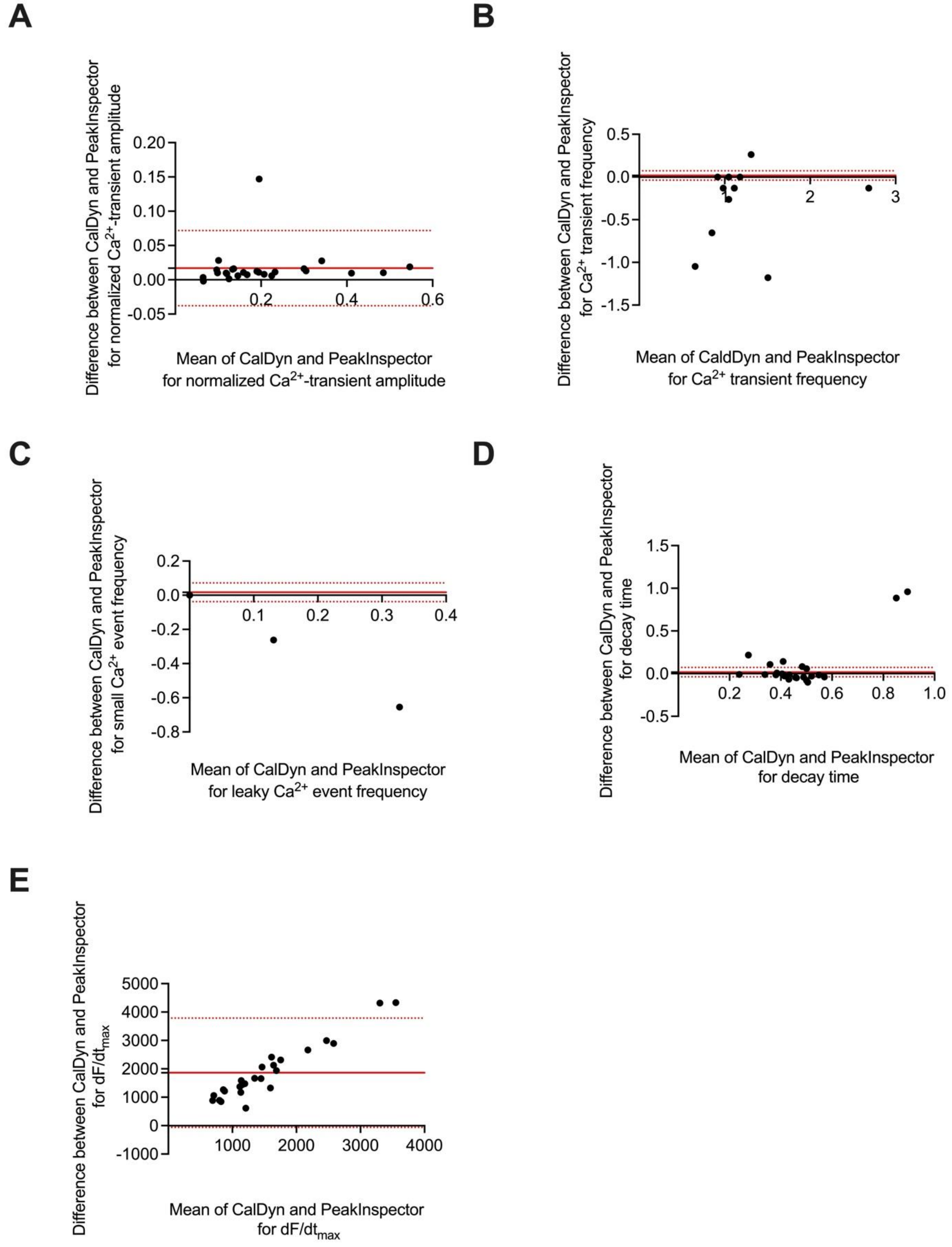

Supplementary Figure 1: Bland–Altman analysis of agreement between CalDyn and PeakInspector for cell-level Ca^{2+}-transient parameters. Differences were calculated as CalDyn minus PeakInspector and plotted against the mean of the measurements obtained using the two methods. (A) Normalized Ca^{2+}-transient amplitude. (B) Ca^{2+}-transient frequency. (C) Small Ca^{2+}-event frequency. (D) Decay time. (E) Maximum Ca^{2+}-transient rise rate, dF/dt_{max}. Each symbol represents one matched cell-level measurement analyzed using both CalDyn and PeakInspector. Peak-level measurements generated by PeakInspector were summarized within each cell before comparison with the corresponding CalDyn cell-level output. The solid red horizontal line indicates the mean paired difference between the methods (bias), the red dotted lines indicate the lower and upper 95% limits of agreement, calculated as the bias ± 1.96 standard deviations of the paired differences, and the solid black horizontal line indicates zero difference. Negative values indicate lower estimates obtained using CalDyn, whereas positive values indicate higher estimates obtained using CalDyn relative to PeakInspector. Numerical estimates of bias, standard deviation of paired differences, and lower and upper 95% limits of agreement are reported in **Table 4**.

Alt text: Five Bland–Altman plots showing CalDyn-minus-PeakInspector differences against the mean of the two methods for matched healthy hiPSC-CMs. The panels show normalized Ca^{2+}-transient amplitude, transient frequency, small-event frequency, decay time, and maximum rise rate. Horizontal lines indicate the mean bias, the lower and upper 95% limits of agreement, and zero difference.

Supplemental Figure 2:

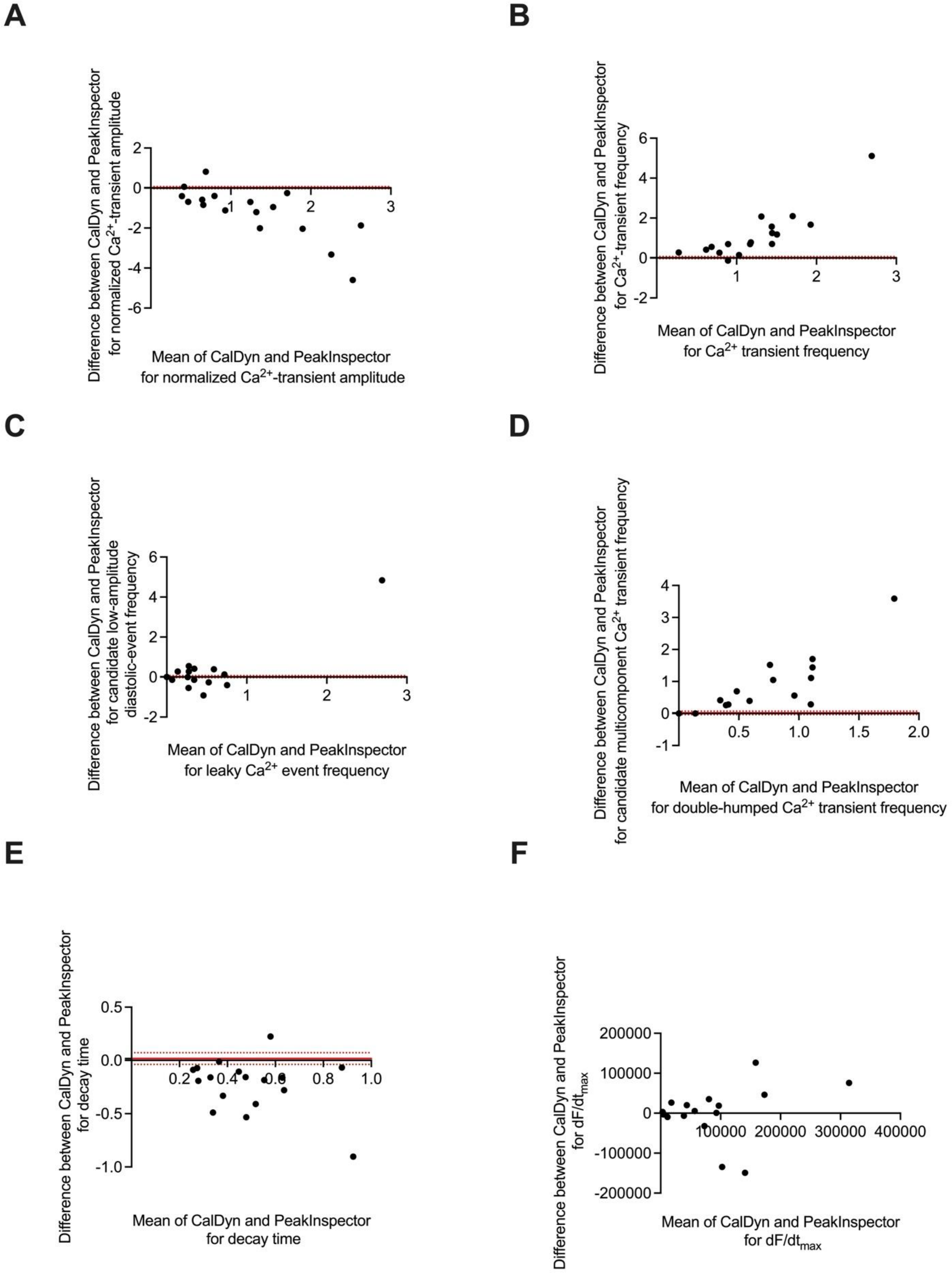

Supplementary Figure 2: Bland–Altman analysis of agreement between CalDyn and PeakInspector for intracellular Ca^{2+}-transient parameters in arrhythmic PMVT hiPSC-CMs. Differences were calculated as CalDyn minus PeakInspector and plotted against the mean of the measurements obtained using the two programs. (A) Normalized Ca^{2+}-transient amplitude. (B) Principal Ca^{2+}-transient frequency. (C) Candidate low-amplitude diastolic-event frequency. (D) Candidate multicomponent Ca^{2+}-transient frequency. (E) Decay time. (F) Maximum Ca^{2+}-transient rise rate, dF/dt_{max}. Each symbol represents one matched recording. The solid red line indicates the mean bias, the red dotted lines indicate the lower and upper 95% limits of agreement, and the solid black line indicates zero difference. Negative values indicate lower estimates obtained using CalDyn, whereas positive values indicate higher estimates obtained using CalDyn relative to PeakInspector. Numerical agreement estimates are reported in **Table 5**.

Alt-text: Six Bland–Altman plots showing CalDyn-minus-PeakInspector differences against the mean of the two programs for matched arrhythmic PMVT hiPSC-CM recordings. The panels show normalized Ca^{2+}-transient amplitude, principal-transient frequency, candidate low-amplitude diastolic-event frequency, candidate multicomponent-transient frequency, decay time, and maximum rise rate. Horizontal lines indicate the mean bias, the lower and upper 95% limits of agreement, and zero difference.

Supplemental Figure 3:

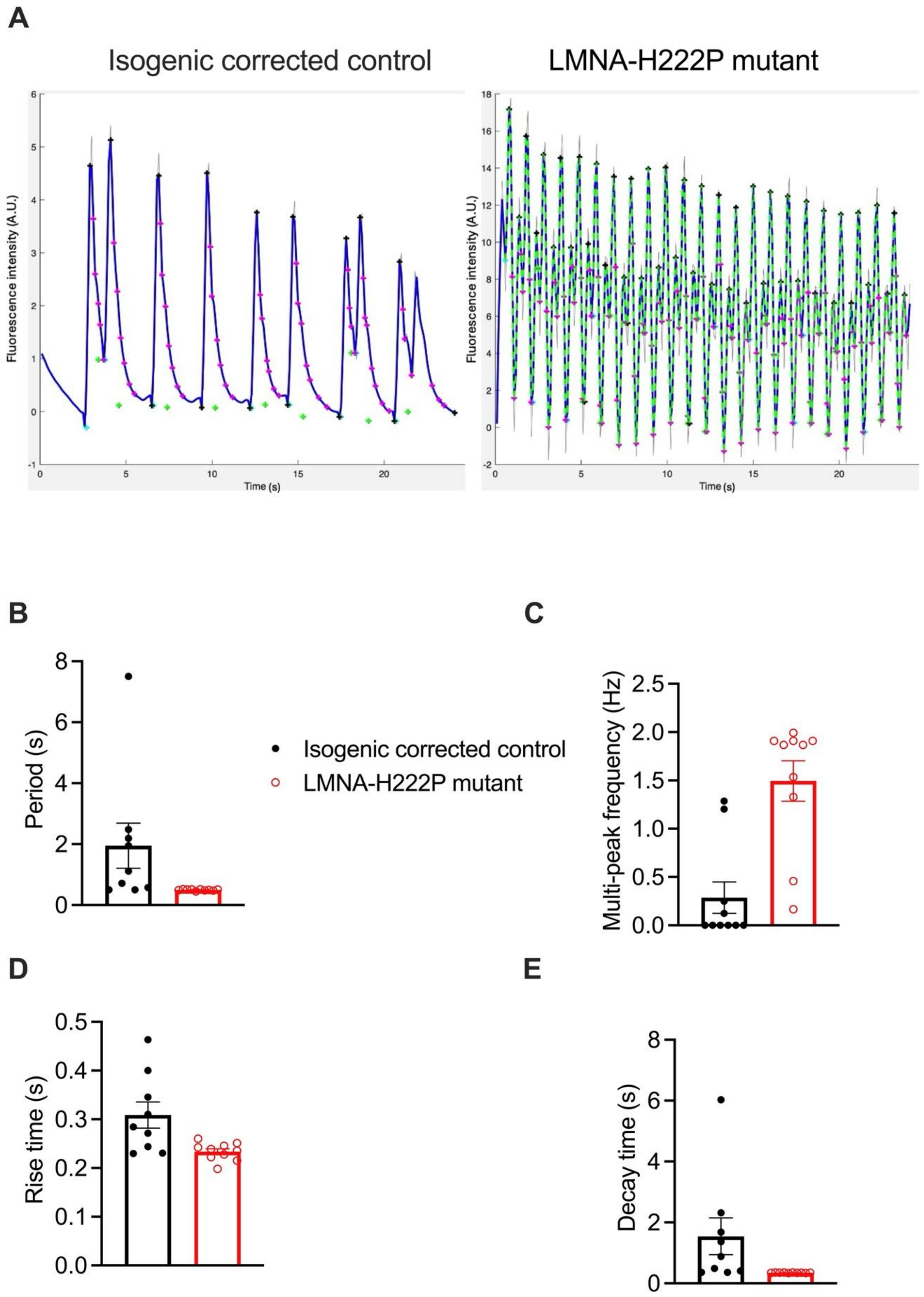

Supplementary Figure 3: Application of CalDyn to Ca^{2+}-transient analysis in LMNA p.H222P and gene-corrected isogenic-control cardiac sheets. (A) Representative intracellular Ca^{2+}-transient recordings from gene-corrected isogenic-control and LMNA p.H222P cardiac sheets analyzed using CalDyn. (B–E) CalDyn-derived inter-transient period (B), multipeak-event frequency (C), rise time (D), and decay time (E). Individual symbols represent 20 regions of interest sampled from four independent cardiac sheets, with 10 ROIs and two sheets analyzed per genotype. ROIs obtained from the same sheet are nested observations and are not independent biological replicates. Bars indicate mean ± SEM. No inferential statistical test was performed because of the limited number of independent cardiac sheets. The data are presented descriptively as an application of CalDyn to a multicellular genetic disease model. ROI, region of interest.

Alt-text: Representative Ca^{2+}-transient traces and ROI-level measurements show inter-transient period, multipeak-event frequency, rise time, and decay time in LMNA p.H222P and gene-corrected isogenic-control cardiac sheets.

Supplemental Figure 4:

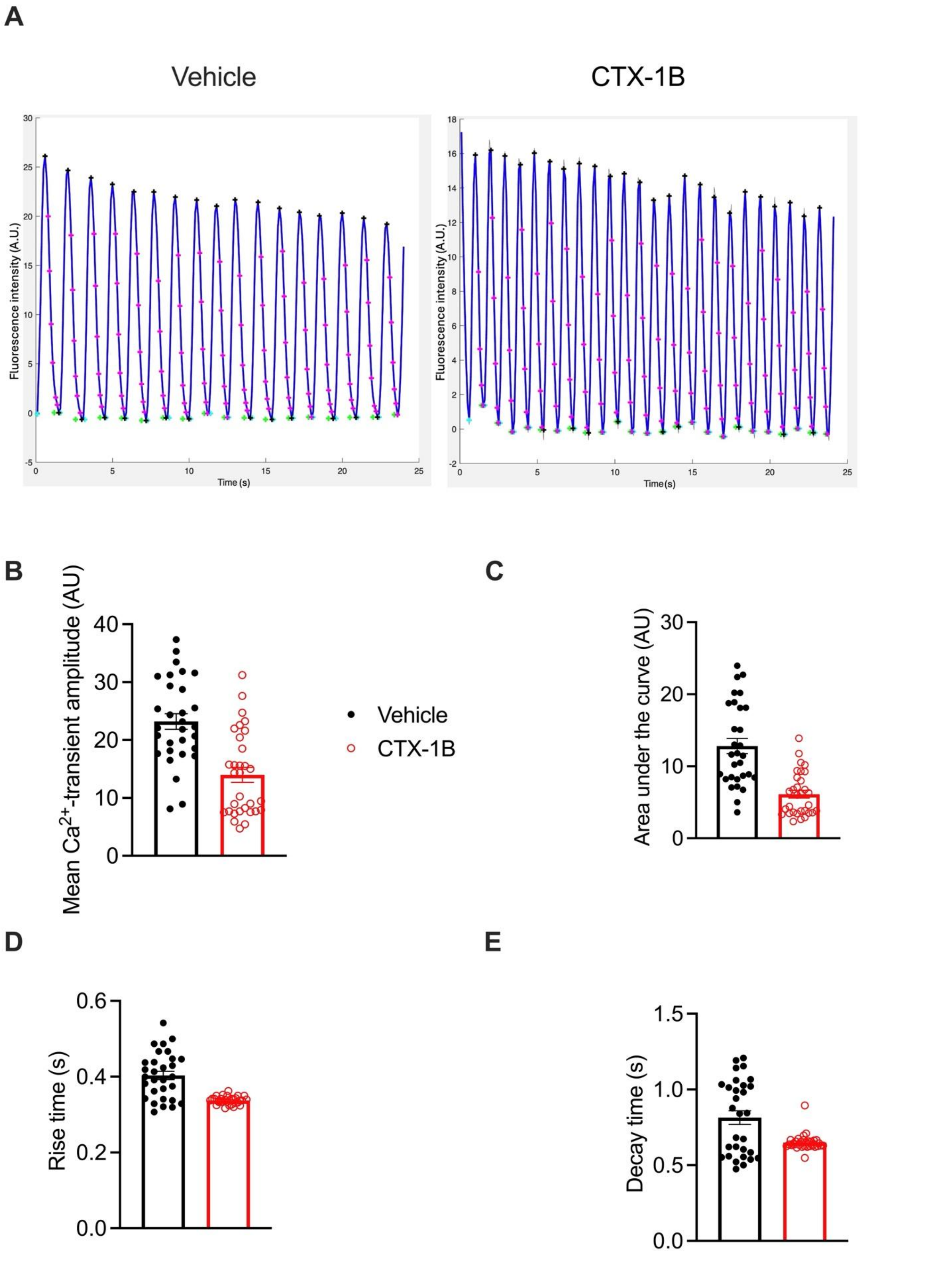

Supplementary Figure 4: Application of CalDyn to calcium-transient analysis in vehicle- and CTX-1B-exposed cardiac sheets. (A) Examples of intracellular calcium-transient recordings from healthy control cardiac sheets exposed to vehicle or 5 nM CTX-1B (acute exposure) and analyzed using CalDyn. (B–E) Calcium-transient fluorescence amplitude, inter-transient period, rise time, and decay time, respectively. Individual symbols represent regions of interest sampled from four independently treated cardiac sheets, including 30 ROIs from two vehicle-treated sheets and 31 ROIs from two CTX-1B-treated sheet. ROIs obtained from the same sheet are nested observations and are not independent biological replicates. Bars indicate mean ± SEM. No inferential statistical test was performed because of the limited number of independent cardiac sheets. The data are presented descriptively as an application of CalDyn to an acute pharmacological perturbation. CTX-1B, Pacific ciguatoxin-1B; ROI, region of interest.

Alt-text: Representative Ca^{2+}-transient traces and ROI-level measurements show amplitude, inter-transient period, rise time, and decay time in vehicle- and 5 nM CTX-1B-exposed cardiac sheets.

Supplemental Figure 5:

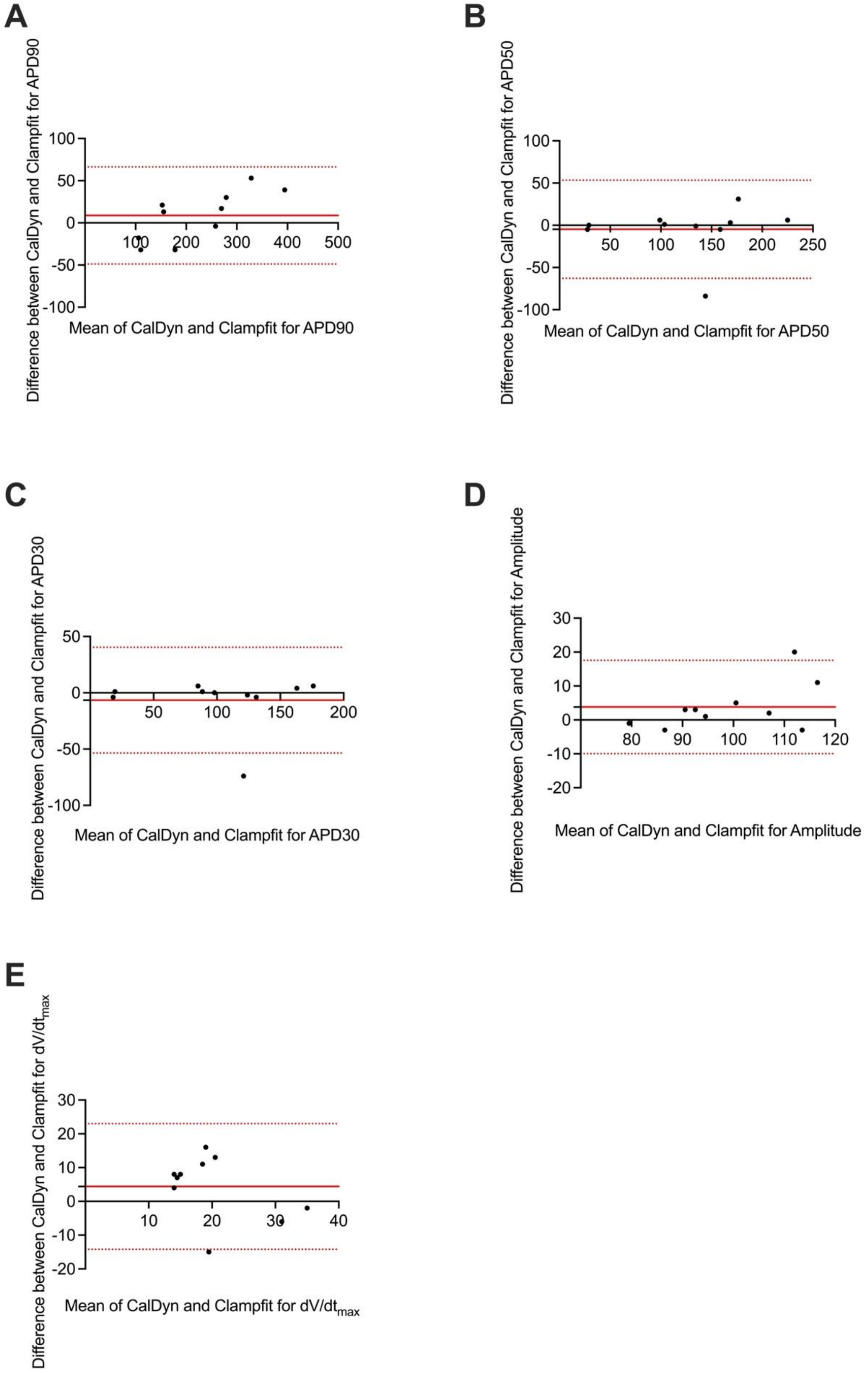

Supplementary Figure 5: Bland–Altman analysis of agreement between CalDyn and Clampfit for action-potential parameters. Differences were calculated as CalDyn minus Clampfit and plotted against the mean of the measurements obtained with the two methods. (A–C) Bland–Altman plots for action-potential duration at 90%, 50%, and 30% repolarization (APD_{90}, APD_{50}, and APD_{30}), respectively. (D) Bland–Altman plot for action-potential amplitude. (E) Bland–Altman plot for maximum action-potential upstroke velocity (dV/dt_{max}). Each symbol represents one matched recording analyzed with both CalDyn and Clampfit. The red solid horizontal line indicates the mean difference between the methods (bias), and the dotted lines indicate the lower and upper 95% limits of agreement, calculated as the bias ± 1.96 standard deviations of the paired differences. Negative values indicate lower estimates obtained with CalDyn, whereas positive values indicate higher estimates obtained with CalDyn relative to Clampfit. Numerical estimates of bias, standard deviation of paired differences, and 95% limits of agreement are reported in **Table 6**.

Alt-text: Five Bland–Altman plots showing CalDyn-minus-Clampfit differences against the mean of the two methods for matched action-potential recordings. The panels show action-potential duration at 90%, 50%, and 30% repolarization, action-potential amplitude, and maximum upstroke velocity. Horizontal lines indicate the mean bias, the lower and upper 95% limits of agreement, and zero difference.

Supplementary Table 1:

Analysis	Dataset	Line/donor	Independent biological preparations	Sheets/recordings	Cells/ROIs or sites	Events analyzed	Experimental unit	Statistical unit	Provenance
Figure 3	Healthy hiPSC-CMs	Healthy control AG08C5 line	3 independent differentiations	One recording per cell	25 cells	7–8 Ca^{2+} transients per cell	Independent differentiation	Cell/recording, nested within differentiation; matched between methods	Previously generated and reanalyzed
Figure 4	PMVT hiPSC-CMs	RyR2-H29D patient-derived hiPSC line	3 independent differentiations	One recording per cell	17 cells	7–8 Ca^{2+} transients per cell	Independent differentiation	Cell/recording, nested within differentiation; matched between methods	Previously published dataset reanalyzed for the present study
Supplementary Figure 3	LMNA cardiac sheets	LMNA p.H222P line and gene-corrected isogenic control	2 independent differentiations	4 cardiac sheets	20 ROIs	isogenic control:0-47 peaks/ROI LMNA mutant: 45-52 peaks/ROI;	Independently generated cardiac sheet, nested within differentiation	Cardiac sheet; ROIs nested within sheet	Previously generated and reanalyzed
Supplementary Figure 4	Vehicle/ CTX-1B cardiac sheets	Healthy control ROMDES line	2 independent differentiations	4 independently treated cardiac sheets	Vehicle: 30 ROIs; CTX-1B: 31 ROIs	Vehicle: 12–31 peaks/ROI; CTX-1B: 19–27 peaks/ROI	Independently treated cardiac sheet, nested within differentiation	Cardiac sheet; ROIs nested within sheet	Previously generated and analyzed for the present study
Figure 7	Current-clamp action potentials	Healthy control AG08C5 line	2 independent differentiations	One recording per cell	10 cells	Approximately 40 APs per cell	Cell	Cell/recording, matched between methods	Newly generated and analyzed for the present study
Figure 8	Optical mapping	Dunkin–Hartley guinea-pig hearts	3 independent hearts	One recording per heart	24 sites	Approximately 4 APs per site	Heart	Heart; mapping sites nested within heart	Previously published dataset reanalyzed for the present study

Supplementary Table 1. Dataset provenance, biological-replication hierarchy, and analytical units used in the principal validation and application experiments. For each analysis, the table reports the biological model, cell line or donor, number of independent biological preparations, number of cardiac sheets or recordings, number of cells, regions of interest (ROIs), or optical-mapping sites, and the number of detected events contributing to the analysis. The experimental unit denotes the independently generated, treated, or sampled biological preparation to which the experimental condition was applied. The statistical unit denotes the level at which observations were entered into the statistical analysis. Cells and recordings obtained from the same differentiation, ROIs obtained from the same cardiac sheet, peaks obtained from the same cell or ROI, and optical-mapping sites obtained from the same heart constitute nested measurements and were not considered independent biological replicates. For paired software comparisons, each cell or recording provided one matched summary value for each analytical method, while its parent differentiation is reported as the higher-level biological replicate. The provenance column

identifies datasets that were newly generated for the present study, reanalyzed from previously reported experiments, or shared with a companion study. AP, action potential; CTX-1B, ciguatoxin-1B; hiPSC-CM, human induced pluripotent stem cell-derived cardiomyocyte; PMVT, polymorphic ventricular tachycardia at rest; ROI, region of interest.

Supplementary Movie S1. Ca^{2+} activity in a gene-corrected isogenic-control cardiac sheet. Representative time-lapse fluorescence recording of spontaneous intracellular Ca^{2+} activity in a cardiac sheet generated from the gene-corrected isogenic-control hiPSC line. Changes in fluorescence reflect the propagation of Ca^{2+} transients across the multicellular cardiac sheet. Fluorescence time series extracted from regions of interest within these recordings were analyzed using CalDyn, as illustrated in Supplementary Figure 3.

Supplementary Movie S2. Ca^{2+} activity in an LMNA p.H222P cardiac sheet. Representative time-lapse fluorescence recording of spontaneous intracellular Ca^{2+} activity in a cardiac sheet generated from the patient-derived LMNA p.H222P hiPSC line. Changes in fluorescence show the spatial and temporal patterns of Ca^{2+} transients across the multicellular cardiac sheet. Fluorescence time series extracted from regions of interest within these recordings were analyzed using CalDyn, as illustrated in Supplementary Figure 3.
